# The Neuronal Circuit of the Dorsal Circadian Clock Neurons in *Drosophila melanogaster*

**DOI:** 10.3389/fphys.2022.886432

**Published:** 2022-04-29

**Authors:** Nils Reinhard, Frank K. Schubert, Enrico Bertolini, Nicolas Hagedorn, Giulia Manoli, Manabu Sekiguchi, Taishi Yoshii, Dirk Rieger, Charlotte Helfrich-Förster

**Affiliations:** ^1^ Julius Maximilian University of Würzburg, Würzburg, Germany; ^2^ Neurobiology and Genetics, Theodor-Boveri-Institute, Biocenter, University of Würzburg, Würzburg, Würzburg, Germany; ^3^ Graduate School of Natural Science and Technology, Okayama University, Okayama, Japan

**Keywords:** circadian clock, *Drosophila melanogaster*, dorsal clock neurons, trans-tango, flybow, neuroanatomy, hemibrain, clock network

## Abstract

*Drosophila*’s dorsal clock neurons (DNs) consist of four clusters (DN_1a_s, DN_1p_s, DN_2_s, and DN_3_s) that largely differ in size. While the DN_1a_s and the DN_2_s encompass only two neurons, the DN_1p_s consist of ∼15 neurons, and the DN_3_s comprise ∼40 neurons per brain hemisphere. In comparison to the well-characterized lateral clock neurons (LNs), the neuroanatomy and function of the DNs are still not clear. Over the past decade, numerous studies have addressed their role in the fly’s circadian system, leading to several sometimes divergent results. Nonetheless, these studies agreed that the DNs are important to fine-tune activity under light and temperature cycles and play essential roles in linking the output from the LNs to downstream neurons that control sleep and metabolism. Here, we used the Flybow system, specific split-GAL4 lines, *trans*-Tango, and the recently published fly connectome (called hemibrain) to describe the morphology of the DNs in greater detail, including their synaptic connections to other clock and non-clock neurons. We show that some DN groups are largely heterogenous. While certain DNs are strongly connected with the LNs, others are mainly output neurons that signal to circuits downstream of the clock. Among the latter are mushroom body neurons, central complex neurons, tubercle bulb neurons, neurosecretory cells in the pars intercerebralis, and other still unidentified partners. This heterogeneity of the DNs may explain some of the conflicting results previously found about their functionality. Most importantly, we identify two putative novel communication centers of the clock network: one fiber bundle in the superior lateral protocerebrum running toward the anterior optic tubercle and one fiber hub in the posterior lateral protocerebrum. Both are invaded by several DNs and LNs and might play an instrumental role in the clock network.

## Introduction

Circadian clocks anticipate the 24-h rhythms on Earth. In animals, a master clock is located in the brain that tunes physiology, metabolism, and behavior to their respective temporal niche. In mammals and insects, this circadian master clock consists of tightly interacting neurons that may fulfill different roles in the clock network based on their neurochemistry, morphology and physiology (Beckwith and Ceriani, 2015; Stengl and Arendt, 2016; Helfrich-Förster, 2017; Herzog et al., 2017; Top and Young, 2018; Rojas et al., 2019; King and Sehgal, 2020; Michel and Meijer, 2020; Mieda, 2020; Ahmad et al., 2021).

**FIGURE 1:**
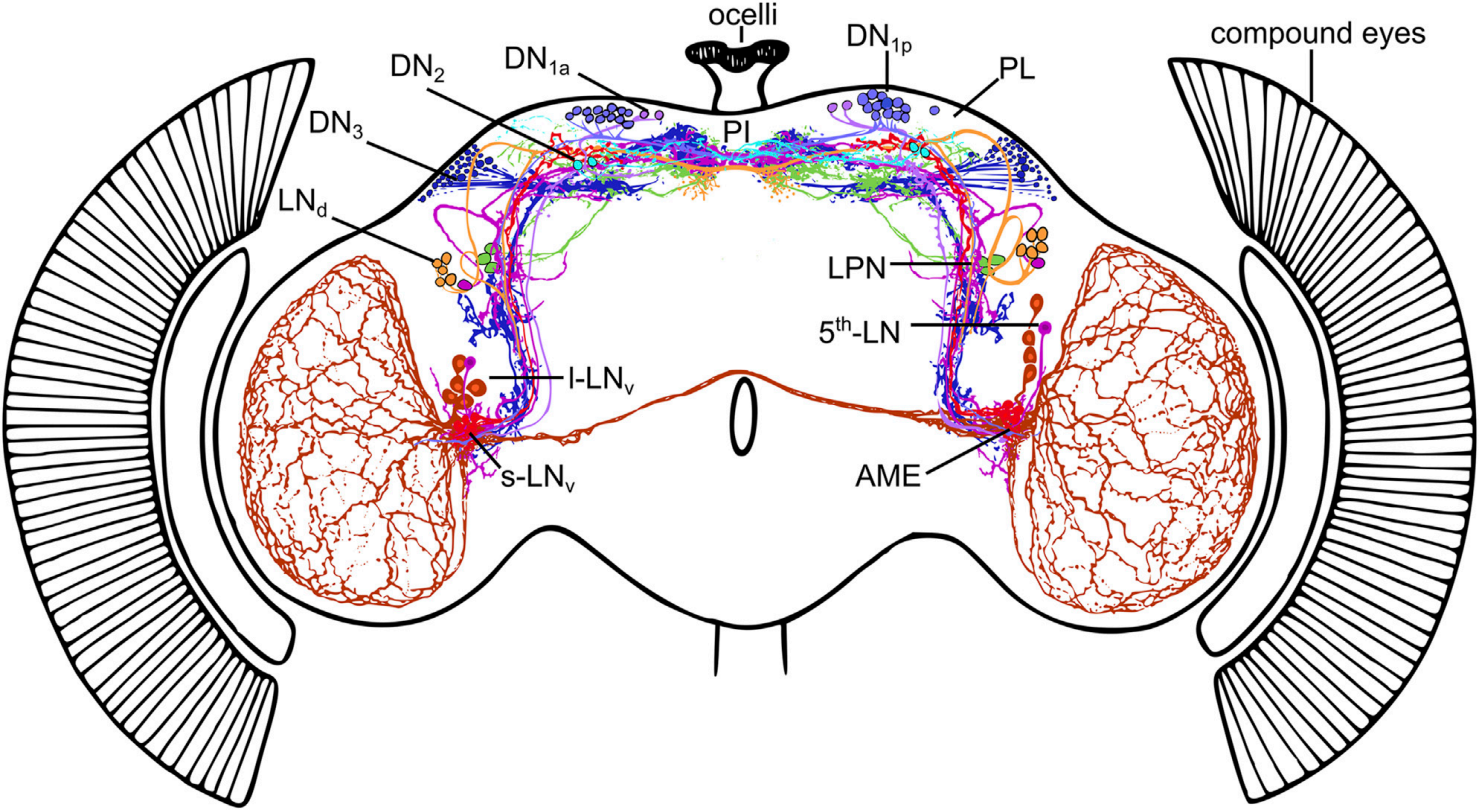
The circadian clock neurons in the brain of *Drosophila melanogaster*. The clock network consists of Lateral Neurons (s-LN_v_s, 5^th^-LN, l-LN_v_s, LN_d_s_,_ and LPNs) and Dorsal Neurons (DN_1a_s, DN_1p_s, DN_2_s, DN_3_s), the neurites of which are highly connected. Many of them send fibers into the dorsal protocerebrum (including the neurosecretory centers in the pars intercerebralis (PI) and pars lateralis (PL)) as well as into the accessory medulla (AME) of both hemispheres, small neuropils at the base of the medulla. The AME can be regarded as the communication center of the clock neurons. Modified from Helfrich-Förster et al., 2007a with data from Schubert et al., 2018 and Reinhard et al., 2022 and this study.

The clock network of *Drosophila melanogaster* consists of only about 150 clock neurons which can be divided according to the location of their cell bodies into five lateral and four dorsal groups. Some of them overlap with their projections in the ventrolateral brain in the so-called accessory medulla (AME) and in the dorsomedial brain, important communication centers of the clock network (Figure 1; Guo et al., 2016, 2018; Chatterjee et al., 2018; Fujiwara et al., 2018; Goda et al., 2018; Schubert et al., 2018; Díaz et al., 2019; Reinhard et al., 2022). So far, most attention has been focused on the lateral neurons in the anterior brain (s-LN_v_s, l-LN_v_s, fifth LN, and LN_d_s) because some of them appear most important for driving rhythmic behavior under constant environmental conditions, in the absence of all Zeitgebers (Ewer et al., 1992; Frisch et al., 1994; Helfrich-Förster et al., 1998; Renn et al., 1999; Grima et al., 2004). More recently, the lateral neurons in the posterior brain (LPNs) have also been studied in detail and found to be involved in the control of sleep and activity patterns in the presence of light-dark or temperature cycles but not under constant environmental conditions (Chen et al., 2016; Díaz et al., 2019; Ni et al., 2019; Reinhard et al., 2022).

The four groups of dorsal neurons (DN_1a_s, DN_1p_s, DN_2_s, and DN_3_s) are the least well characterized. Like the LPNs, the DNs appear to be less important in controlling rhythmic behavior under constant environmental conditions. Nevertheless, they appear to fine-tune behavioral rhythmicity under light and temperature cycles (Miyasako et al., 2007; Picot et al., 2009; Yoshii et al., 2009; Zhang L. et al., 2010, Zhang et al., 2010 Y.; Harper et al., 2016; Chatterjee et al., 2018; Yadlapalli et al., 2018; Goda et al., 2019; Ni et al., 2019; Lamaze and Stanewsky, 2020). Furthermore, these clock neurons play essential roles in the output from the core clock network to downstream neurons. For example, they participate in the control of sleep and metabolism (Cavanaugh et al., 2014; Kunst et al., 2014; Barber et al., 2016; Guo et al., 2016, 2018; Ni et al., 2019)

Here, we used a combination of GAL4, GAL80, and newly generated split-GAL4 transgenic lines (Sekiguchi et al., 2020) to address specific groups of dorsal clock neurons that were not accessible so far. These drivers combined with the Flybow system (Hadjieconomou et al., 2011; Shimosako et al., 2014; Schubert et al., 2018) and with other tools, including *trans*-Tango (Talay et al., 2017), and electron microscopic data from the hemibrain (Scheffer et al., 2020) allowed us to characterize the anatomy and the synaptic connections of some DNs in greater detail. We will discuss our results in the light of recent physiological and behavioral studies that provide insights into the functions of the DNs.

## Materials and Methods

### Fly Strains, Husbandry, and Crossings

Unless stated otherwise, fly strains used in this study were reared on standard cornmeal medium with yeast at 25 ± 0.2°C and 60 ± 5% relative humidity under light-dark cycles (LD) of 12:12 h. All fly lines used are described in Supplementary Table S1 (including references).

To reveal the anatomy of specific clock neurons, *GAL4* or split-GAL4 lines were crossed to a membrane-bound GFP reporter (10xUAS-myr::GFP) or to a cytoplasmic myc-tagged GFP reporter (20UAS-6xGFP, Supplementary Table S1). They were co-stained with antibodies against the clock protein Period (PER) and different antibodies against several neuropeptides to verify their neurochemistry (*see* immunohistochemical procedure below). To reveal the post- and presynaptic sites of the neurons of interest, we crossed the *GAL4*-drivers to UAS-DenMark::mCherry and UAS-nSyb::EGFP, respectively (Supplementary Table S1).

A driver stock for usage with the Flybow system (*see* below) was built by balancing the *GAL4*-lines listed in Table 1 and crossing them to *y w;hs-mFlp5* ^*MH12*^ */CyO; TM2/TM6B* or to *y w; GlaBc/CyO;hs-mFlp* ^*5MH3*^ */TM6B* depending on which chromosome the *GAL4* insertion was located. Experimental flies were obtained by crossing the balanced *GAL4/hs-mFlp5* lines to either *hs-Flp* ^*1*^ *;+;FB2.0B* ^*49b*^ or to *hs-Flp* *^1^* *;FB2.0B* ^*260b*^;+ (Supplementary Table S1).

### Flybow

For labeling individual clock neurons we used the revised multicolor Flybow system with the *Flybow2.0B* reporter construct, which carries an FRT-site flanked stop-codon upstream of the fluorescent protein sequences (Shimosako et al., 2014). The stop codon can be removed by Flp-recombination, induced by a heat shock-promotor controlled hs-Flp DNA-recombinase. Experimental flies were kept at 18°C (18 ± 0.2°C and 60 ± 5% relative humidity with a LD cycle of 12:12) to prevent uncontrolled Flippase recombination events. Three heat shocks (37°C) of 45–60 min were applied on three consecutive days to induce Flippase-recombinase activity at different larval and pupal stages until the clock network was fully developed, which appears to be the case in late pupal stages (Helfrich-Förster et al., 2007a). Adult flies (4–5 days after eclosion) were dissected and the brains were immunolabeled with anti-GFP, anti-mCherry, and anti-nc82 antibody-solution, as described in Schubert *et al.* (2018).

### *Trans*-Tango

To reveal putative post-synaptic partners of selected clock neurons, we applied the *trans*-Tango technique (Talay et al., 2017) in combination with new clock driver lines (Sekiguchi et al., 2020). The crosses were reared at 25°C and the progeny transferred at 18°C after eclosion for several days, to obtain an accumulation of the post-synaptic signal. In general, we sampled flies after 17, 28, 30, 34, and 53 days at 18°C and found that the post-synaptic signal strength increases significantly with time. Presynaptic neurons were revealed by anti-GFP and postsynaptic neurons by anti-Hemagglutinin (HA) antibody staining (*see* below and Supplementary Table S2).

### Immunocytochemistry

Fluorescent immunocytochemistry was performed according to the protocol described in Schubert *et al.* (2018). In brief, male flies were fixed as whole animals for 2–3 h in 4% paraformaldehyde after they have been entrained to LD12:12 cycles for 4–5 days after eclosion. For anti-PER staining, fixation was performed 1 h before lights-on (ZT23), since PER accumulation is maximal at this time (Zerr et al., 1990). For the other antibody staining, flies were fixed around ZT3. For staining against Cryptochrome (CRY), the flies were kept several days under constant darkness to allow CRY to accumulate. After dissecting the brains in phosphate-buffered saline (pH 7.4) they were incubated in 5% normal goat serum in PBT 0.5% overnight and then incubated in the primary antibody solution for ∼2 days. Fluorescent tagged secondary antibodies were applied for at least 3 h. All used antibodies are listed in Supplementary Table S2. The immunostained brains were aligned on a specimen slide, embedded in Vectashield 1000 mounting medium (Vector Laboratories, Burlingame, CA, United States), and stored at 4°C until scanning. If not otherwise stated at least 10 different brains were stained for each antibody or antibody combination.

### Confocal Microscopy and Image Processing

Fluorescence protein expression and antibody staining were visualized and scanned with a Leica TCS SP8 or a Leica SPE confocal microscope (Leica Microsystems, Wetzlar, Germany), Leica SP8 was equipped with hybrid detectors, a photon multiplier tube, and a white light laser for excitation, using the laser and detector settings as described in Shimosako *et al.* (2014). Leica SPE was equipped with a photomultiplier tube and 488, 532, and 635 nm solid-state lasers for excitation.

We used a 20-fold glycerol immersion objective (HC PL APO, Leica Microsystems, Wetzlar Germany) for whole-mount scans and obtained confocal stacks with 2 µm z-step size and 1024 × 1024 pixels. For a more detailed view, we used a 63-fold glycerol objective (HC PL APO, Leica Microsystems, Wetzlar Germany) and scanned the brains with a resolution of 2048 × 2048 pixels and a z-resolution of 1 µm. All focal planes were scanned three to four times and the frames were averaged to reduce background noise. The hybrid detectors of SP8 were used with photon counting mode and each focal plane was scanned and accumulated four times. The obtained confocal stacks were maximum projected and analyzed with Fiji ImageJ (Schindelin et al., 2012). Besides contrast and brightness, no further manipulations were done to the confocal images. For displaying the depth information in maximum projections we used the plug-in “Z-stack Depth Color Code” (v.0.0.2) for Fiji. For the reconstruction of the DN_2_s_,_ we used the simple neurite tracer plug-in (Arshadi et al., 2021). The reconstructions were visualized using the natverse libraries (v 0.2.4, Bates et al., 2020) for RStudio (v 1.3.1093). The anti-nc82 staining (anti-bruchpilot) was used to align our stacks to the Janelia Farm Research Campus standard brain (JFRC2) by using the computational morphometry tool kit graphical user interface plug-In for Fiji (CMTK GUI, Rohlfing and Maurer, 2003; Jefferis et al., 2007). We compared our anatomical results with the results gained in the hemibrain of a single female fly (Scheffer et al., 2020) and the reconstructions of the DN_1a_s by Marin *et al.* (2020) available for the full adult female brain (FAFB, Zheng et al., 2018).

### Comparison With Electron Microscopic Reconstruction Data

For a comparison of our findings with the reconstructed neurons of the electron microscopic data set from the hemibrain and the FAFB, we used the natverse libraries (v 0.2.4, Bates et al., 2020) and hemibrainr package (v.0.5.0, Bates and Jefferis, 2022) for R (v. 4.0.5) *via* RStudio (v 1.3.1093). The neuron-specific information was obtained from the hemibrain dataset (Scheffer et al., 2020, v 1.2.1) of the neuprint server (neuprint.janelia.org). For the FAFB dataset (Zheng et al., 2018) the reconstructions were obtained from the Virtual Fly Brain CATMAID server (fafb.catmaid.virtualflybrain.org). The annotation of the neurons was used as a reference and the identity of the neurons was verified by their morphology and the orientation in the brain if possible. For morphological comparisons, the neurons and brain regions of the hemibrain were transformed into the JRC 2018F template space (Bogovic et al., 2020) using xform_brain (nat.templatebrains v 1.0). When different neuron types were shown in the same brain, the neurons were additionally mirrored to the other hemisphere. Connectivity data was visualized using the ggplot2 package (v3.3.5, Wickham, 2016).

## Results

To facilitate understanding, we describe the morphology and synaptic connectivity of the different dorsal neurons in the context of the existing literature and, where appropriate, discuss the reasons for differences between studies.

### The DN_1a_s

The DN_1a_s express PER from the first larval instar onward (Kaneko et al., 1997). They are glutamatergic (Hamasaka et al., 2007; Collins et al., 2012) and in adult flies, they express additionally the neuropeptides IPNamide (Shafer et al., 2006) and CChamide1 (Fujiwara et al., 2018). Furthermore, they express the intracellular blue-light receptor Cryptochrome (CRY) (Yoshii et al., 2008). We addressed the two DN_1a_s with Flybow using the *R16C05-GAL4* driver (Pfeiffer et al., 2008). Using this method, we were able to trace individual DN_1a_s in 30 brains, whereas in 11 brains only a single DN_1a_ was labeled and could be analyzed at the single-cell level. In addition, the two DN_1a_s were stained together in the DN_1a_-specific split-GAL4 line *R43D05-p65.AD; R93B11-DBD* (Sekiguchi et al., 2020). In the following, we will describe the morphology of the DN_1a_s according to the results of both staining methods compared to the reconstructions at the electron microscopic level (Supplementary Video S1).

**FIGURE 2:**
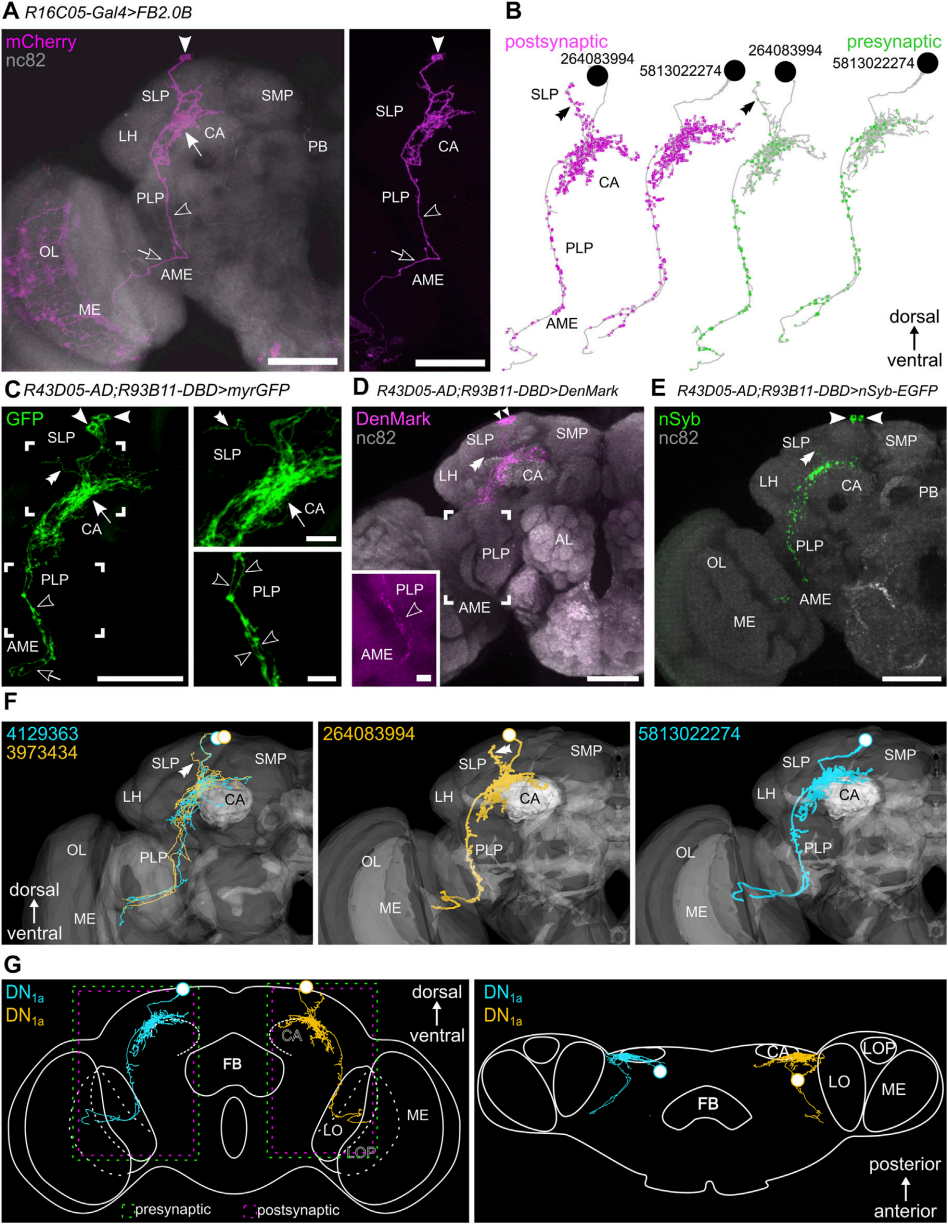
The morphology of the two DN_1a_s. **(A)** Flybow-reporter expression (mCherrry, magenta) driven by *R16C05-GAL4* and anti-nc82 neuropil staining (gray). Only one of the two DN_1a_s is labeled together with several small cells in the optic lobe. A single fiber of this neuron projects from the SLP through the ventromedial lateral horn (LH) and the posterior lateral protocerebrum (PLP) into the accessory medulla (AME) and further toward the surface of the medulla (ME) (open arrow). The arrow points to its dense arborizations around the mushroom body calyx (CA). **(B)** Postsynaptic (magenta) and presynaptic (green) sites of the two DN_1a_s (revealed in the hemibrain) are distributed over the entire neurons. **(C)** Morphology of both DN_1a_s visualized in the highly DN_1a_ specific split-GAL4 line with membrane-bound GFP (maximal projection of 47 confocal planes). Both DN_1a_s project to the AME (open arrow and arrowhead) and only differ by a faint anteriorly projecting fiber in the SLP (double arrowhead, *see* also inset). The insets show magnifications of the arborizations close to the CA and the PLP. **(D)** Staining against the DenMark reporter (mCherry) which visualizes the dendritic fibers of the DN_1a_s. The inset shows a magnification of the ventrally running fibers towards the AME with a maximal projection of only 15 confocal planes for clarification. **(E)** GFP staining of the nSyb-EGFP fusion protein that visualizes the axonal terminals of the DN_1a_s. **(F)** Reconstructions of the two DN_1a_s on the electron microscopic level derived from the FAFB (Marin et al., 2020) and the hemibrain (Scheffer et al., 2020) The double arrowhead highlights the anterior running arm of one of the two DN_1a_s (yellow). **(G)** Schematic overview of the DN_1a_ morphology from a frontal (left) and dorsal (right) view. The rectangles with dashed lines in the left picture represent post- and presynaptic sites as revealed by DenMark and nSyb staining, respectively. LOP, lobula plate; LO, lobula; CA, mushroom body calyx; SLP, superior lateral protocerebrum; SMP, superior medial protocerebrum; LH, lateral horn; AL, antennal lobes; PB, protocerebral bridge; OL, optic lobes; FB, fan-shaped body; ME, medulla; Scale bars represent 50 and 10 µm in detail views.

The cell bodies of both DN_1a_s are located in the anterior superior cell body rind dorsal to the superior lateral protocerebrum (SLP) (Shafer et al., 2006). Initially, the DN_1a_s project ventrally along the surface of the SLP into the posterior-most part of the SLP. The projections of both cells bifurcate at the level of the posterior boundary between the lateral horn (LH) and the SLP and invade the posterior-most part of the LH. Here, the neurites of both DN_1a_s branch extensively and innervate the ventromedial region of the posterior LH (Figure 2C), together with the s-LN_v_ terminals. Both neurons send projections medially that terminate dorsally to the Kenyon cells of the mushroom bodies in the lateral and dorsal accessory calyx (CA, Figures 2A,C, arrow). Further projections run ventrally to the accessory medulla (AME, Figure 2, open arrowhead). Concerning the shape and extent of arborizations in the posterior SLP and LH, the two neurons differed slightly. The varicose arborizations of one DN_1a_ appeared slightly more restricted around the CA whereas the other DN_1a_ showed less dense projections, extending slightly more medially in the SLP as well as more ventrally in the posterior LH. The biggest difference between the two DN_1a_s represented the single neurite of the neuron with the slightly broader arborization pattern, reaching from the dense ramifications around the CA in the dorsal SLP (Figure 2 double arrowhead). In roughly one-third of the DN_1a_s analyzed with Flybow the projections to the AME were not visible. However, the DN_1a_-specific split-GAL4 line revealed that both DN_1a_s project ventrally (Figure 2C open arrowhead). This projection ran parallel to the neurites of the Pigment Dispersing Factor (PDF)-positive s-LN_v_s until it reached and joined the posterior optic commissure (Figures 2A,C, open arrow). Along with this major fiber bundle, the ventral DN_1a_ projections invaded the ipsilateral AME and often ran onto the surface or along the anteromedial edge of the medulla (Figures 2A,C). Due to the vast overlap of their arborizations in most parts, distinguishing the two neurons in the polarity staining was difficult. We observed signals of the post- and presynaptic markers in the posterior ventromedial LH, probably originating from both DN_1a_s. The presynaptic vesicles in the projection to the AME were consistently and strongly labeled (nSyb::EGFP), whereas only a faint DenMark (TLN::mCherry) signal (postsynaptic marker) was found in these neurites (Figures 2D,E).

The reconstructions of the hemibrain largely support our observations and the two annotated DN_1a_s (#264083994 and #5813022274) have the same characteristic arborization pattern as described above (Figure 2F). The reconstructions by Marin *et al.* (2020) using the FAFB dataset (Figure 2F, neuron #4129363 and #3973434) support additionally our observation that the DN_1a_s differ in the neurite reaching in the dorsal SLP (Figure 2F double arrowhead). Output synapses (presynaptic terminals) were present throughout the neurites, but less densely in the one fiber of DN_1a_ #264083994 that projects dorsally into the SLP. This dorsally projecting fiber carried mainly input synapses (postsynaptic terminals) (Figure 2B double arrowhead).

### Synaptic Partners of the DN_1a_s

Both DN_1a_s appear strongly linked to the s-LN_v_s. The neurites of both neuron types have been shown to largely overlap with each other (Fujiwara et al., 2018), an observation that we confirm with our study. The DN_1a_s overlap with the terminals of the s-LN_v_s in the SLP and with the dendrites of the s-LN_v_s in the AME. Also functionally, the DN_1a_s and the s-LN_v_s are connected as was shown by previous work: the DN_1a_s signal *via* glutamate and the neuropeptide CCHamide1 to the s-LN_v_s (Hamasaka et al., 2007; Collins et al., 2012; Fujiwara et al., 2018; Song et al., 2021) while the s-LN_v_s signal *via* glycine and PDF to the DN_1a_s (Shafer et al., 2008; Yoshii et al., 2009; Frenkel et al., 2017). To further investigate this mutual synaptic connection and to identify synaptic outputs of the DN_1a_s to other downstream neurons we performed *trans*-Tango and visualized the results by anti-HA in flies of different ages (17, 28, 30 days).

**FIGURE 3:**
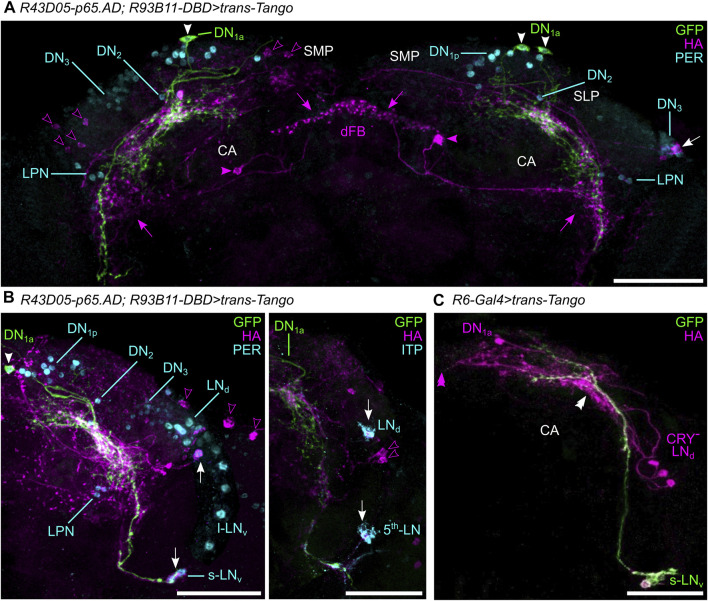
Neurons pre- and postsynaptic to the DN_1a_s revealed by *trans*-Tango. **(A)** Maximal projections of 30 confocal planes depicting a frontal view of the anterior part of the dorsal protocerebrum with the presynaptic DN_1a_s marked in green (green fluorescent protein, GFP), their postsynaptic partners in magenta (hemagglutinin, HA), and other clock neurons marked in cyan (labeled with anti-PER). One to two DN_3_ clock neurons were labeled by HA and are consequently postsynaptic to the DN_1a_s (white arrow). HA additionally labeled unknown postsynaptic neurons (open magenta arrowheads) in the superior medial (SMP) and the superior lateral protocerebrum (SLP). Further, a neuron of the eighth layer of the fan-shaped body (FB) was labeled as postsynaptic to the DN_1a_s (magenta arrowheads). It innervated not only the dorsal FB (dFB) but also the posterior lateral protocerebrum at about the height of the LPNs (magenta arrows). In this region, a network of fibers postsynaptic of the DN_1a_s is found that is adjacent to the dense varicose fibers of the DN_1a_s. **(B)** Maximal projection of 40 confocal planes depicting frontal views of the right lateral protocerebrum with the presynaptic DN_1a_s labeled in green and their postsynaptic partners in magenta. In the left picture, all clock neurons are labeled by anti-PER in cyan, whereas in the right picture the ITP-positive clock neurons (LN_d_ and fifth LN) are labeled in cyan. White arrows point to clock neurons that are postsynaptic to the DN_1a_s, Two s-LN_v_s and one LN_d_ (left) and the ITP-positive fifth LN and LN_d_ (right). Note that in the brain shown to the left the l-LN_v_s and the fifth LN_d_ are located quite dorsally and cannot be distinguished from each other. The LN_d_s are also located more dorsally than usually and overlap with the DN_3_s. **(C)** Frontal view of the lateral protocerebrum with the presynaptic s-LN_v_s marked by GFP and their postsynaptic partners in magenta (maximal projection of 30 confocal planes). The s-LN_v_ appear to have synaptic contacts with at least one DN_1a_ and the three CRY-negative LN_d_s. Since the CRY-negative LN_d_s do not project contralaterally to the other hemisphere (Schubert et al., 2018) the midline of the brain (magenta double arrowhead) is free of marked fibers. The white double arrowhead marks the putative area of synaptic contacts between the s-LN_v_ and the DN_1a_/LN_d_. CA, calyx of the mushroom bodies. The scale bars represent 50 µm.

When driving *trans*-Tango in the DN_1a_s, several clock neurons (PER-positive) were co-labeled by the HA-antibody (Figures 3A,B): one to two DN_3_s, the Ion transport peptide (ITP)-positive LN_d_ and fifth LN, one to four s-LN_v_s (Figures 3A,B, white arrows) and sometimes weakly one to four l-LN_v_s as already shown by Song *et al.* (2021). Also, the DN_1a_s themselves were labeled in some brains. In addition, one characteristic neuron of the dorsal fan-shaped body (dFB) was marked by anti-HA. Its cell body was located in the posterior brain (Figure 3A magenta arrowheads) with neurites running into the eighth layer of the FB and laterally toward a dense fiber hub close to the LPNs, in which several clock neurons arborize and the DN_1a_s contribute few fibers (Figure 3A, magenta arrows; *see* also later). Further non-clock neurons were labeled in the lateral, dorsolateral and posterior dorsal brain. These were located close to the LN_d_s, DN_3_s, and DN_1p_s, respectively, but were not double-labeled by anti-PER (Figures 3A,B, open magenta arrowheads). While the DN_3_s and most of the LNs were already stained in young flies, the neuron projecting to the dFB was only marked in older flies.

When driving *trans*-Tango in the s-LN_v_s, we found that at least one DN_1a_ is postsynaptic to the s-LN_v_s (Figure 3C). In addition, the three CRY-negative LN_d_s were marked, indicating that they are also postsynaptic to the s-LN_v_s.

**FIGURE 4:**
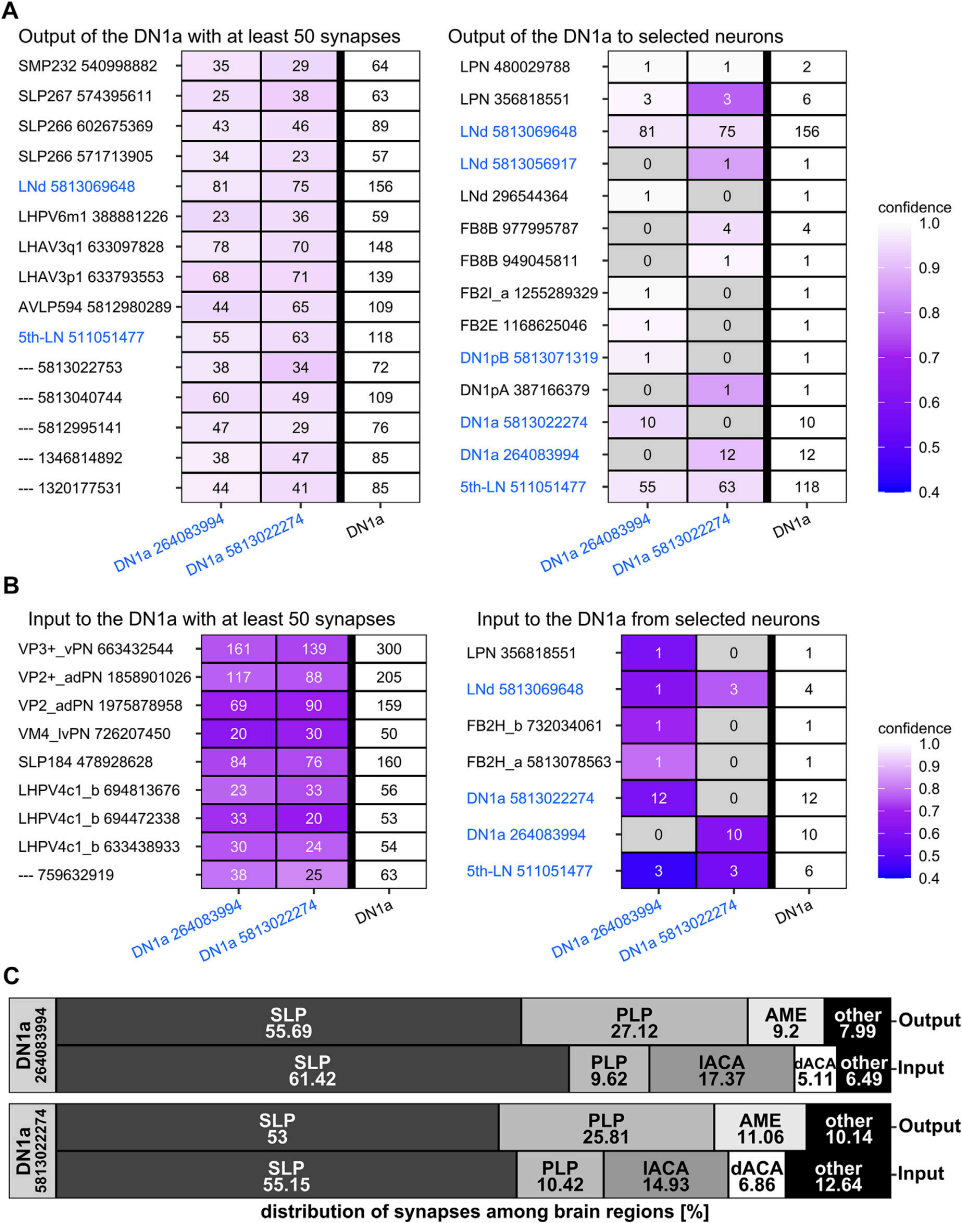
Synaptic contacts of the two DN_1a_s revealed from the hemibrain. **(A)** Postsynaptic partners of the two DN_1a_s with at least 50 synapses (left panel) and output connections to selected partners such as the clock neurons and the fan-shaped body (FB) neurons (right panel). CRY-positive clock neurons are highlighted in blue. The DN_1a_s give strong output to neurons with cell bodies located in the superior lateral protocerebrum (SLP) or the lateral horn (LH). Among these are the ITP positive lateral neurons (LN_d_ and fifth LN). In addition, the two DN_1a_s form synapses with each other, but they have relatively few output synapses to the other clock neurons, including a neuron arborizing in the eighth layer of the FB. **(B)** Presynaptic partners of the two DN_1a_s with at least 50 synapses and selected presynaptic partners. The DN_1a_s get most input from temperature sensing projection neurons of the antennal lobes (VP3+_vPN, VP2+_adPN, VP2_adPN) and considerable input from neurons with cell bodies in the LH and SLP. Only little input comes from the clock neurons. **(C)** Synapse distribution of the two DN_1a_s among the brain regions. The DN_1a_s form about 50% of their input and output synapses in the SLP. In the second place are output synapses in the PLP, an area from which they also get input, although to a lower amount. Furthermore, they give output to the AME but do not receive input worth mentioning from there. Instead, they receive considerable input from the lateral and dorsal accessory calyx (lACA, dACA). As can be expected from their similar morphology, the two DN_1a_s largely coincide in synapse distribution and synaptic partners.

The *trans-*Tango staining was rather consistent with the presynaptic (output) sites of the DN_1a_s revealed on the ultrastructural level in the hemibrain (Figure 4A). Most output synapses were found in the superior protocerebrum (SLP and SMP) to the ITP- and CRY-positive LN_d_ and the fifth LN, but no outputs were found to the s-LN_v_s (Figure 4A) and very few to DN_3_-like neurons (*see* below). The virtual lack of output to the DN_3_s is understandable because these neurons are not unequivocally annotated in the hemibrain, but it is less clear why the s-LN_v_s are not among the listed postsynaptic partners of the DN_1a_s. Most probably, they form only a small amount of synapses with these clock cells that have been overlooked. The same might be true for the input synapses from the s-LN_v_s that we detected in our *trans-*Tango staining. The s-LN_v_s are not listed as input partners of the DN_1a_s in the hemibrain (Figure 4B). Nevertheless, input from the s-LN_v_s to the DN_1a_s was also revealed in a recent functional study, strongly indicating that our observation is valid (Song et al., 2021).

As expected from the slightly different morphology of the two DN_1a_s, DN_1a_ #264083994 with the predominantly dendritic fiber in the SLP received slightly more input from neurons located in this brain region. Otherwise, the two DN_1a_s show large similarities in their synaptic connections (Figure 4C) and seem to signal to each other (Figure 4B).

While the main DN_1a_ input and output synapses are formed in the SLP and posterior lateral protocerebrum (PLP), their neurites in the AME appear mainly presynaptic. The DN_1a_s get especially strong input (20% of their input synapses) in the dorsal and lateral accessory calyces that are located adjacent to the SLP (Figure 4C). Most of this input appears to come from temperature sensing neurons in the antennal lobes (VP3+_vPN/VP2+_adPN as well as VP2_adPN, Figure 4B) as was also revealed by Marin *et al.* (2020).

### The DN_1p_s

The DN_1p_s are a demonstrably heterogeneous group in respect to their neurochemistry and morphology. About half of the approximately 15 cells per brain hemisphere express CRY and the PDF receptor (PDFR) (Shafer et al., 2008; Yoshii et al., 2008; Im and Taghert, 2010). Five to six of the CRY-positive DN_1p_s are positive for glutamate (Hamasaka et al., 2007; Guo et al., 2016) and the Diuretic Hormone 31 (DH31, Kunst et al., 2014; Goda et al., 2016) and at least four of these CRY-/DH31-positive neurons express additionally Allatostatin C (AstC, Díaz et al., 2019; Zhang et al., 2021) and CNMamide (Jung et al., 2014; Abruzzi et al., 2017; Jin et al., 2021; Zhang et al., 2021). As recently shown, four AstC-positive DN_1p_s additionally express the receptors for PDF and DH31 (Goda et al., 2018; Lin et al., 2022). The neurochemistry of the CRY-/PDFR-negative DN_1p_s is so far unknown.

It is not surprising that the DN_1p_s differ also in their morphology. The latter has been described in detail in three studies coming from different labs (Chatterjee et al., 2018; Guo et al., 2018; Lamaze et al., 2018). These studies used combinations of different *GAL4*, *LexA*, *GAL80,* and split-GAL4 lines to address different subgroups of the DN_1p_s and stochastic flip outs to label single neurons. The most important line, which we used also in our study, is the *Clk4.1M-GAL4* line. It targets about 10 out of the 15 DN_1p_s comprising the majority (∼6 out of 7) of the CRY-positive neurons plus ∼4 CRY-negative cells (Figure 2; Zhang Y. et al., 2010, Zhang et al., 2010 L.; Chatterjee et al., 2018). The *R18H11-GAL4* line from the FlyLight library collection (Pfeiffer et al., 2008) originates from the gene coding for PDFR and targets only the five to six CRY-/PDFR-positive neurons that are additionally glutamate- and DH31-positive (Kunst et al., 2014; Goda et al., 2016; Guo et al., 2016, 2018; Chatterjee et al., 2018). In contrast, the *R18H11-LexA* line additionally drives in few CRY-/PDFR-negative DN_1p_s (Chatterjee et al., 2018; Guo et al., 2018), presumably because PDFR expression is not completely absent in the PDFR-negative characterized neurons, but only largely reduced, so that it is still revealed by strong drivers. Nevertheless, none of the used lines targets all DN_1p_s, so additional types of DN_1p_s may exist (especially CRY-negative ones) that have not yet been characterized.

Previous studies have principally identified two main types of DN_1p_s: One type remaining ipsilaterally and projecting to the anterior optic tubercle (AOTU) (the so-called anterior projecting dorsal neurons), while the other type crosses the midline of the brain through the pars intercerebralis but lacks projections to the AOTU (Chatterjee et al., 2018; Lamaze et al., 2018). According to Chatterjee *et al.* (2018), the DN_1p_s projecting to the AOTU express CRY, whereas it is unclear which DN_1p_s of the other type are CRY-positive.

**FIGURE 5:**
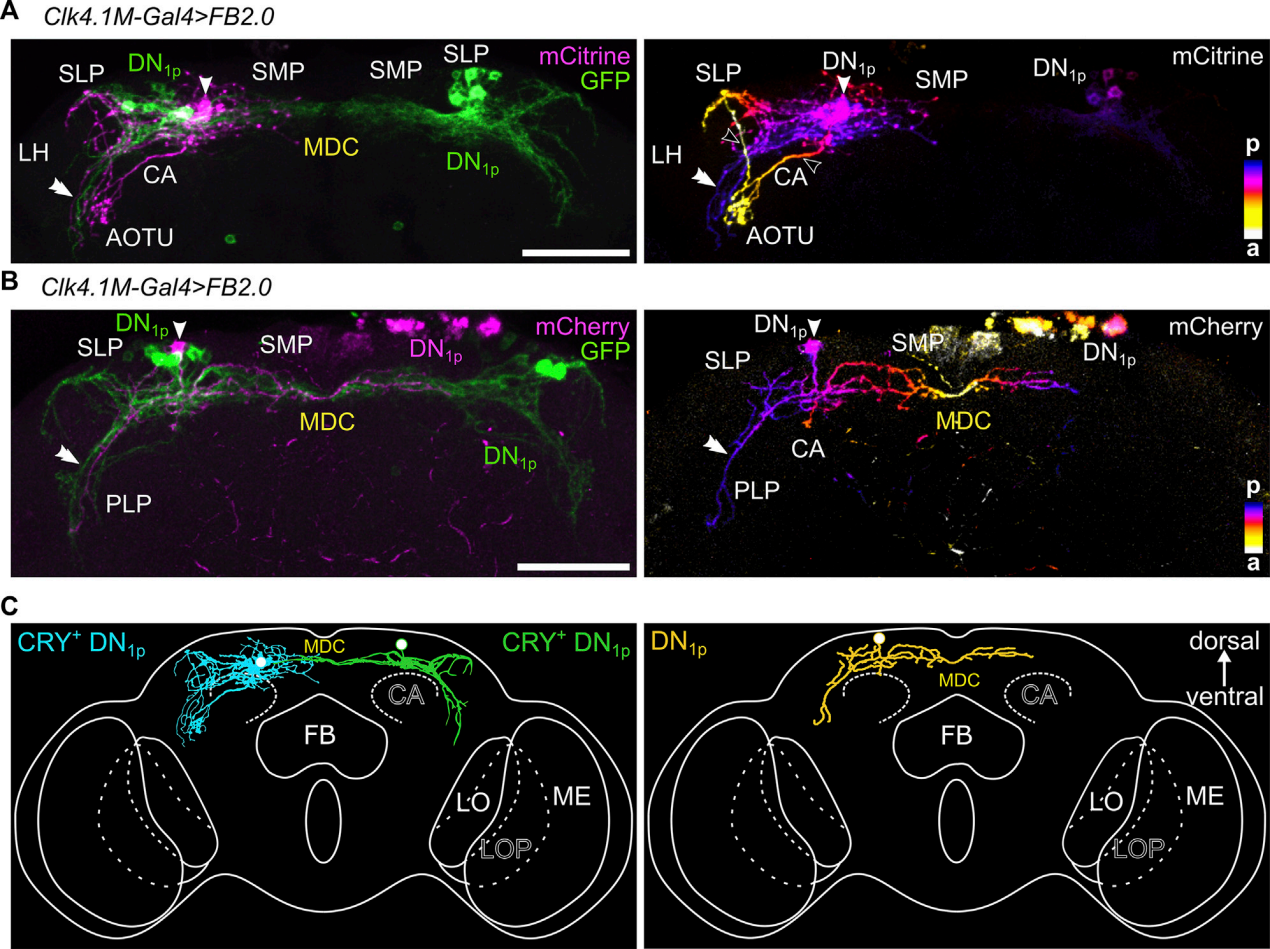
Different morphological subtypes within the DN_1p_s as revealed by Flybow staining. **(A**,**B)** *Clk4.1M-GAL4* driven Flybow-reporter expression in different DN_1p_s. The left panels show the original Flybow-reporter expression in which an individually labeled neuron (magenta, arrowhead) expresses mCitrine or mCherry, while several other DN_1p_s express the default GFP reporter (green). The right panels depict the single DN_1p_s that are marked in magenta in the left panels with color-coded depth information (anterior (a) in yellow colors, posterior (p) in blue colors as shown in the color code bars in the right bottom of each figure). **(A)** mCitrine marks a single neuron with arborizations in the superior lateral protocerebrum (SLP). Two fibers (open arrowheads) run anterior to the lateral anterior optic tubercle (AOTU), where they arborize prominently. Another fiber (double arrowhead) remains in the posterior brain and runs ventrally following the s-LN_v_ projections. **(B)** Single DN_1p_ expressing mCherry (magenta) among four other GFP expressing DN_1p_s. The single DN_1p_ has a prominent fiber following the path of the s-LN_v_s in the ipsilateral posterior lateral protocerebrum (PLP) and crosses the dorsoventral midline of the brain through the middle dorsal commissure (MDC). No arborizations in the anterior SLP are visible. **(C)** Schematic depiction of the three DN_1p_ subtypes identified by Flybow. SMP, superior medial protocerebrum; LH, lateral horn; PLP, posterior lateral protocerebrum; ME, medulla; LO, lobula; LOP, lobula plate; FB, fan-shaped body. Scale bars represent 50 μm.

We analyzed the DN_1p_s of 22 *Clk4.1M-GAL4* brains in combination with the Flybow-reporter to reveal potential differences in the morphology of the DN_1p_s. As it turned out, the modified Flp-recombinase had not been activated sufficiently, and only four brains showed individually labeled neurons. Nevertheless, we were able to identify three different types of DN_1p_ subgroups. All DN_1p_ cell bodies were located in the dorsal posterior cell body rind innervating the posterior superior medial protocerebrum (SMP) and SLP, as well as the posterior ventromedial LH and dorsal parts of the PLP, where they followed the s-LN_v_ projections in ventral direction, although no fibers reached the AME (Figure 5).

A single labeled DN_1p_ largely resembled the previously described CRY-positive neurons that invade the AOTU and form pronounced varicosities in this region (Figure 5A, called a-DN_1p_ in Lamaze et al., 2018). The fibers of this neuron formed two loops running from posterior to anterior and terminated in the AOTU (open arrowheads). One loop ran around the SLP and the second one ventrally on the surface of the superior clamp (SCL) and the inferior clamp until both converged shortly before the AOTU. In addition, fibers from this neuron remained posterior and followed the s-LN_v_ projections in the PLP (double arrowhead) for a short distance toward the AME. Most significantly, all neurites of this neuron remained ipsilaterally and did not cross the midline of the brain in the middle dorsal commissure (MDC). A similar neuron is described in Figures 1C,D of Lamaze *et al.* (2018) and called anteriorly projecting DN_1p_ (a-DN_1p_).

The other labeled DN_1p_s appeared to belong to the second type of DN_1p_s described by Lamaze *et al.* (2018), Chatterjee *et al.* (2018), and Guo *et al.* (2018). These neurons were called vc-DN_1p__neurons by Lamaze et al. (2018) and sent fibers through the MDC to the contralateral SMP and on the ipsilateral side, their projections in the PLP appeared to reach slightly further toward the AME than that of the first DN_1p_ type (Figure 5B). We could identify two different subtypes with this projection pattern. One neuron of the first subtype was individually labeled (Figure 5B right and yellow neuron in Figure 5C), whereas the presence of the second subtype (Figure 5C green) was concluded from the samples with varying numbers of visible cells. Fibers of the second subtype started to form a loop around the SLP as was typical for the two CRY-positive DN_1p_s described above, but they did not complete this loop and did not reach the AOTU (green neuron in Figure 5C). A very similar neuron (called vc-DN_1p_) is described in Figure 1C of Lamaze *et al.* (2018). Furthermore, similar DN_1p_s were found in Jin *et al.* (2021; Figure 5E in Jin et al.) and Zhang *et al.* (2021; Figure 3C in Zhang et al.) among the CNMamide-positive neurons. Since the latter are CRY-positive, we assume that this type of DN_1p_ is CRY-positive. In case of the yellow colored neuron in Figure 5C, we do not know about its CRY expression. Therefore, no statement about CRY is made. The same applies for all further statements regarding the DN_1p_s.

**FIGURE 6:**
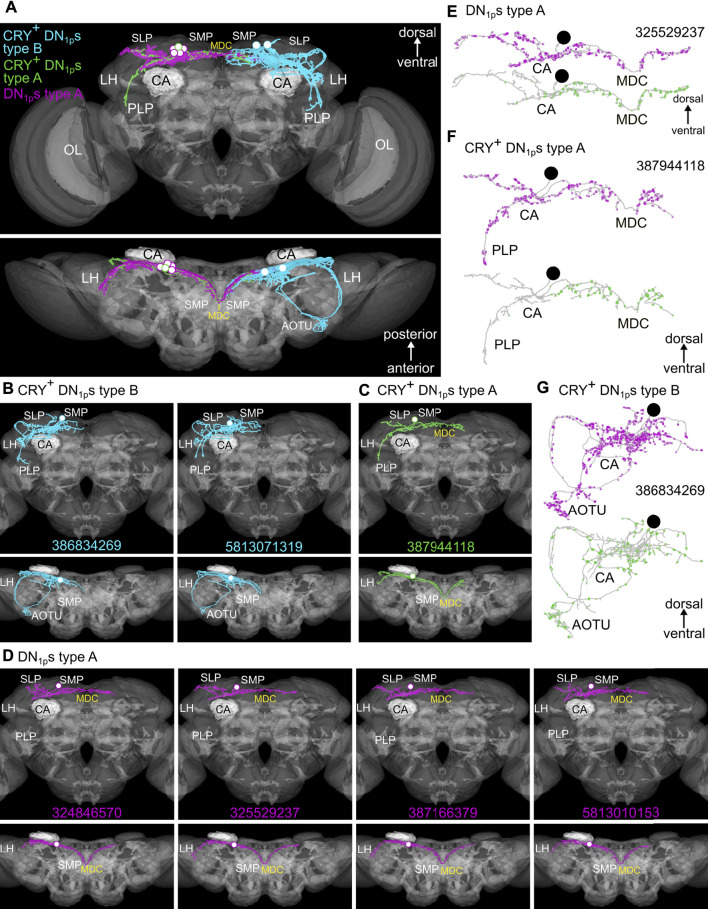
The different morphologies of the seven DN_1p_s annotated in the hemibrain. **(A)** Standard brain (JRC 2018F) showing the three different morphological subtypes among the seven DN_1p_s annotated in the hemibrain. The presumably CRY-positive neurons are depicted in cyan and green, while the still undefined DN_1p_s are shown in magenta. **(B)** Detailed representation of the two Type B CRY-positive DN_1p_s in ventral-dorsal (top) and anterior-posterior (bottom) orientation. Both neurons arborize in the superior lateral protocerebrum (SLP), the superior medial protocerebrum (SMP), and project ventrally to the posterior lateral protocerebrum (PLP). Additionally, they send two fibers to the anterior optic tubercle (AOTU) each of them forming a loop that runs on the dorsal and ventral surface of the SLP, respectively (compare with **G**). **(C)** Representation of the type A CRY-positive DN_1p_ (#387944118). The projections of this neuron remain mostly posterior. In the SLP, this DN_1p_ also sends one fiber toward the AOTU but does not reach it. In further contrast to the two Type B CRY-positive DN_1p_s neurons, it projects *via* the middle dorsal commissure (MDC) to the contralateral superior medial protocerebrum (SMP). **(D)** Representation of four type A DN_1p_s with still undefined nature. These neurons are morphologically similar to the type A CRY-positive DN_1p_ and cross the midline in the MDC, but they remain completely in the SLP and SMP and do not send fibers to the PLP. **(E**–**G)** postsynaptic (input, magenta) and presynaptic (output, green) sites of the three different DN_1p_ subtypes. **(E**,**F)** Type A DN_1p_s receive input all over their neurites but restrict output to the ipsilateral and contralateral SMP. **(G)** Type B DN_1p_s receive input and give output all over their neurites. CA, mushroom body calyx; LH, lateral horn; OL, optic lobes.

**FIGURE 7:**
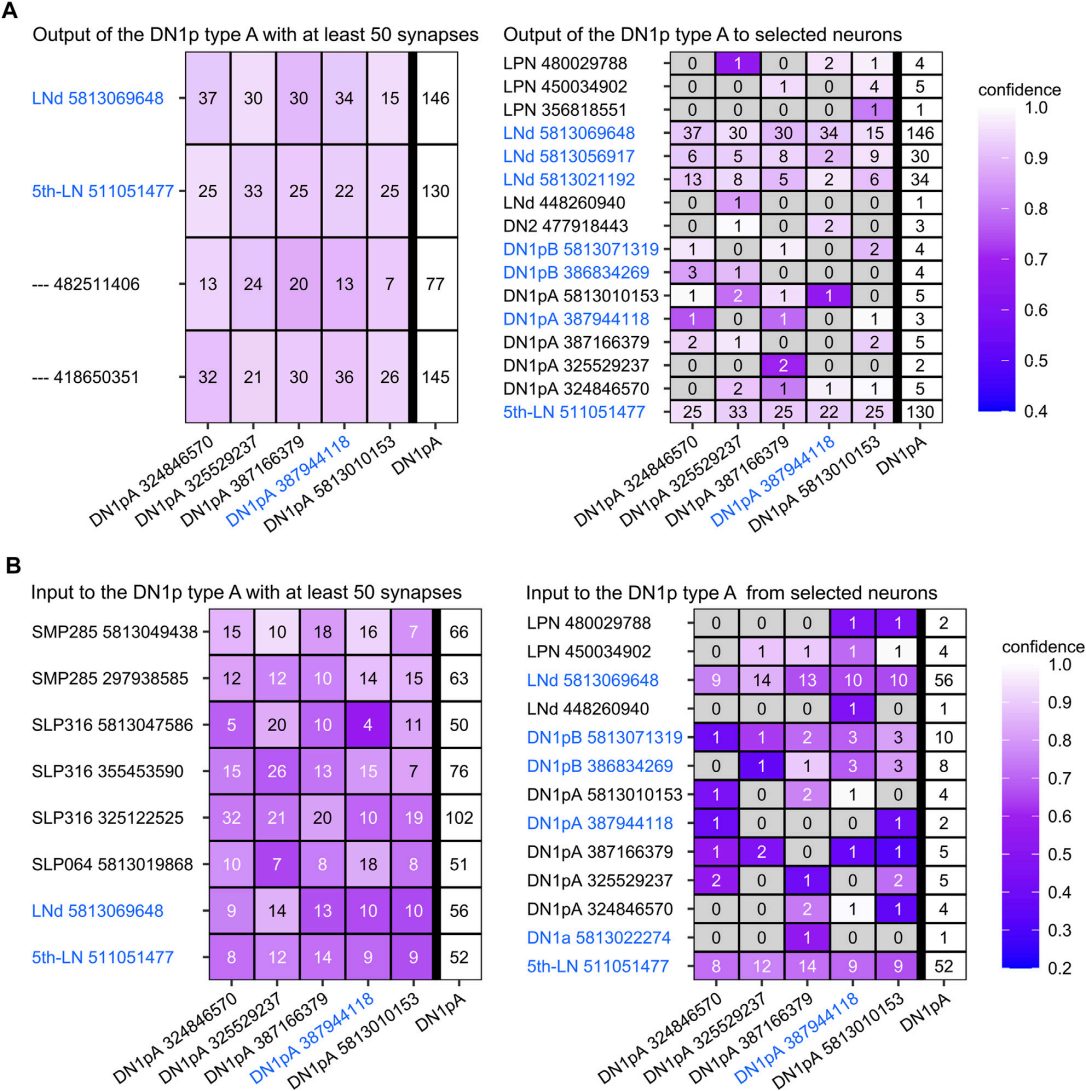
Synaptic partners of the five annotated Type A DN_1p_s from the hemibrain. **(A)** Postsynaptic partners of the Type A DN_1p_s with at least 50 synapses (left panel) and clock neurons as postsynaptic partners (right panel). CRY-positive clock neurons are in blue letters. The Type A DN_1p_s have relatively few postsynaptic partners with more than 50 synapses. Most interestingly the ITP positive lateral neurons (LN_d_ and fifth LN) are among these few neurons. More generally, the type A DN_1p_s are signaling to all three CRY-positive LN_d_s although the synaptic strength of the connections is weaker for the ITP negative ones. Only very few output synapses are established with the other clock neurons. **(B)** Presynaptic partners of the type A DN_1p_s with at least 50 synapses (left panel) and selected clock neurons as presynaptic partners (right panel). The type A DN_1p_s receive most input from neurons with their cell body located in the superior lateral protocerebrum (SLP) as well as in the superior medial protocerebrum (SMP). Among these neurons are again the ITP positive lateral neurons (LN_d_ and fifth LN). Input from other clock neurons is minor, just two of the type A DN_1p_s get weak input from the CRY-positive DN_1p_s. The only for sure CRY-positive Type A DN_1p_ (#387944118, green in Figure 6) is among these two neurons.

**FIGURE 8:**
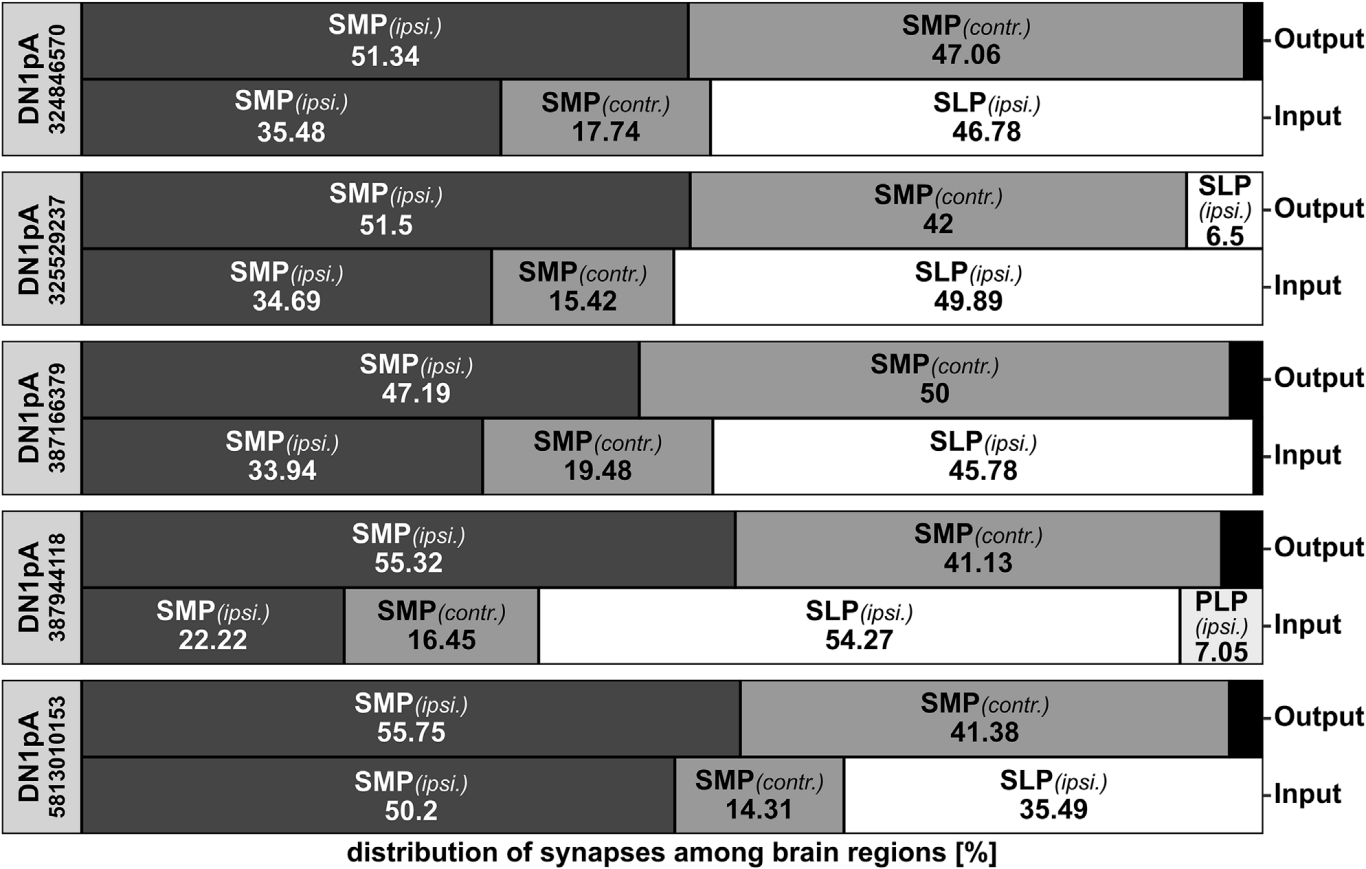
Distribution of the synapses of the five annotated hemibrain type A DN_1p_s among the brain regions. The type A DN_1p_s restrict their output mainly to the ipsilateral (ipsi.) and contralateral (contr.) superior medial protocerebrum (SMP) while they get about 50% of their input additionally from the ipsilateral superior lateral protocerebrum (SLP). The CRY-positive DN_1p_ of type A (#387944118, green in Figure 6) that sends fibers to the posterior lateral protocerebrum (PLP) is the only neuron that receives input from there. In addition, this neuron receives most input from the SLP, to which it sends a dendritic fiber following the loop around the SLP toward the AOTU.

Seven DN_1p_s are annotated in the hemibrain, which were again divided into two types (called A and B in the hemibrain) (Figures 6–8 and Supplementary Video S2). The two type B neurons (cyan in Figure 6) resemble CRY-positive neurons described above, which remain ipsilateral and project into the AOTU. The five type A neurons all project contralaterally and do not enter the AOTU (green and magenta in Figure 6). Among these, the DN_1p_ depicted in green closely resembles the just described CRY-/CNMamide-positive DN_1p_s that send fibers into the anterior SLP and follow the s-LN_v_ projections in the PLP several micrometers (green in Figure 6). The other four type A neurons do not show any fibers following the s-LN_v_ projections in the PLP, while the fibers extending toward the anterior SLP were generally present (magenta in Figure 6). The DN_1p_ neuron type that is not annotated in the hemibrain is depicted in Figure 5C (yellow). This neuron completely lacks the anterior running fibers but shows prominent neurites following the s-LN_v_ projections in the PLP. A similar neuron is described by Lamaze *et al.* (2018; vc-DN_1p_ neuron in Figure 1D) making us confident that this is a true subtype.

#### Synapses and Putative Synaptic Partners of the DN_1p_s

Previous studies used diverse GAL4 lines to perform syt-GFP (Guo et al., 2016, 2018; Lamaze et al., 2018) and DenMark staining (Chatterjee et al., 2018; Guo et al., 2018; Lamaze et al., 2018) and revealed putative presynaptic output sites of the DN_1p_s in the SMP close to the pars intercerebralis and in the AOTU, to which the CRY-positive DN_1p_s project (Guo et al., 2018; Lamaze et al., 2018). Chatterjee *et al.* (2018) found that the characteristic anterior projecting loops of the CRY-positive DN_1p_s around the SLP are not only presynaptic but also dendritic.

**FIGURE 9:**
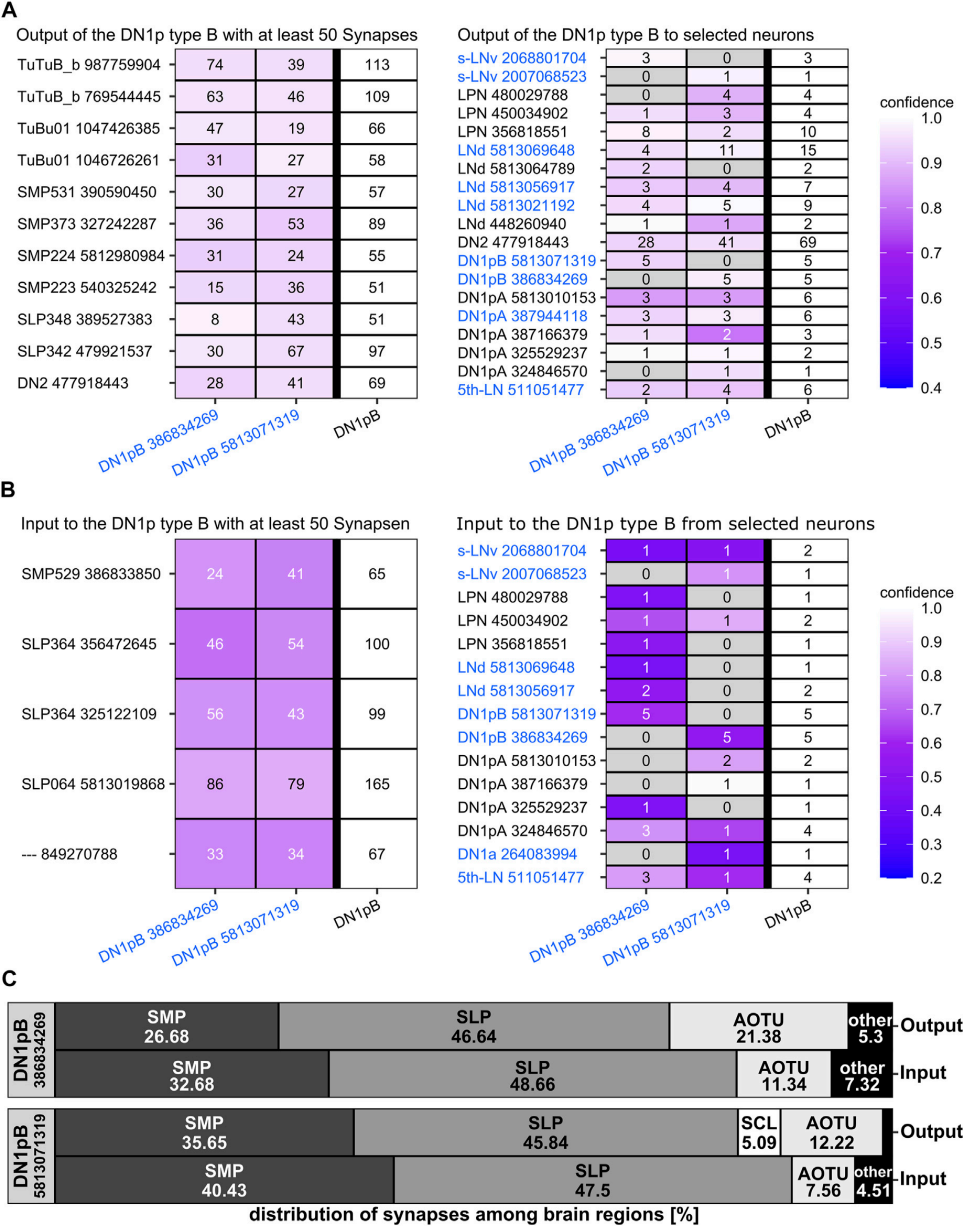
Synaptic partners of the two annotated CRY-positive type B DN_1p_s from the hemibrain. **(A)** Postsynaptic partners of the CRY-positive type B DN_1p_s with at least 50 synapses and postsynaptic clock neurons. As expected, the DN_1p_s project on many tubercle bulb neurons (TuBu) of the anterior optic tubercle (AOTU) and to tubercle-tubercle neurons (TuTuB) which are connecting the AOTUs of both hemispheres. Additionally, neurons with their cell bodies in the superior lateral protocerebrum (SLP) as well as in the superior medial protocerebrum (SMP) get strong input by the CRY-positive type B DN_1p_s. Further, they give strong input to one DN_2_ as the only clock neuron which is getting strong input from the type B CRY-positive DN_1p_s. Other clock neurons like the LPN and the type A DN_1p_s get only weak input from the CRY-positive DN_1p_s. **(B)** As the CRY-positive DN_1p_s give output to neurons of the SMP and SLP they also do get strong input by neurons from these brain regions. Interestingly they do not seem to receive synaptic input from the clock network. **(C)** Distribution of the input and output synapses of the CRY-positive DN_1p_s among the brain regions. The neurons form most of their synapses in the SMP, AOTU, and SLP with most synapses in the latter. The number of input and output synapses are similar in the different brain regions, but the two DN_1p_s slightly differ in the percentage of output synapses in the SMP and AOTU. DN_1p_ #5813071319 has more input and output in the SMP and less input and output in the AOTU than DN_1p_ #386834269. In addition, DN_1p_ #5813071319 is the only so far annotated DN_1p_ that has output in the superior clamp (SCL). Note that the two Type B DN_1p_s do not receive significant input from the PLP, although they send fibers into this region.

We used the connectome data of the hemibrain (Scheffer et al., 2020) to reveal the input and output sites of the DN_1p_s and could confirm the results of Chatterjee *et al.* (2018) and specify the results of the other studies. While the input and output sites of the CRY-positive DN_1p_s (type B neurons) are equally distributed across the entire surface of the neurons, the type A DN_1p_s show output sites mainly in the SMP, while they receive input along the whole neuron (Figures 6E–G, Figure 8, Figure 9). This is true for all type A neurons independent of the subtype (Figure 8).

*Trans*-Tango staining by Guo *et al.* (2018) revealed some LN_d_s and tubercle bulb neurons as possible postsynaptic partners of the DN_1p_s, as well as weak signals in some DN_3_s. Here, we used again the connectivity data from the hemibrain (Scheffer et al., 2020) to investigate the pre-and postsynaptic partners of the DN_1p_s in more detail. Since we found a strong difference between the CRY-positive type B and the more heterogenous type A DN_1p_s, we analyzed the two groups separately (Figures 7–9). The type A DN_1p_s show mainly output to CRY-positive clock neurons with the strongest synaptic output to the ITP and CRY-positive LN_d_ and fifth LN (Figure 7A). Other clock neurons are only contacted with very few synapses and likewise the output to non-clock neurons is generally weak. The connection to the ITP and CRY-positive LN_d_ and the fifth LN appears reciprocal since type A DN_1p_s also receive strong input from these two neurons (Figure 7B). Otherwise, the type A DN_1p_s get little input from the clock network (Figure 7B).

As already shown by Guo *et al.* (2018), the CRY-positive type B DN_1p_s signal to tubercle bulb neurons as well as to tubercle-tubercle neurons which connect both AOTUs (Figure 9A). Additionally, we found that they signal strongly to at least one DN_2_ (Figure 9A), whereas their synaptic contact with other clock neurons is rather weak. Their weak contact within the clock network also concerns the ITP- and CRY-positive LN_d_ and fifth LN, which have strong reciprocal synaptic connections with the Type A DN_1p_s (Figure 9A). There is also very little synaptic input from the clock network to the CRY-positive type B DN_1p_s (Figure 9B).

### The DN_2_s

The DN_2_s consist of only two neurons that have been described for the first time 25 years ago (Kaneko et al., 1997). Their importance in the clock network is highlighted by their early appearance during larval development, together with the s-LN_v_s, the fifth LN, and the DN_1a_s, but their function and fine anatomy are yet unclear. The somata of the DN_2_s are located near the dorsal terminals of the s-LN_v_s and do not seem to express CRY (Benito et al., 2008; Yoshii et al., 2008). The arborizations of both DN_2_s appear to remain in the dorsal protocerebrum and cross the midline of the brain through the pars intercerebralis, to terminate close to the cell bodies of their contralateral counterparts (Helfrich-Förster, 2005). However, due to the wide expression of the clock drivers available (i.e. *Clk856-GAL4*, Gummadova et al., 2009) and the vast overlap of the fibers of other clock neurons in the SLP, it was impossible to finely trace the neurites of the DN_2_s so far.

We used the *Clk206-GAL4* driver in combination with *cry-GAL80* to restrict the expression of Flybow exclusively to the CRY-negative clock neurons, which comprise the DN_2_s. Unfortunately, GAL80 was not sufficient to repress *GAL4* expression in the CRY-positive s-LN_v_s and DN_1a_s, except for the fifth LN. Consequently, none of the 17 brains analyzed contained individually labeled DN_2_s. Nonetheless, since all additionally marked neurons have been described at single-cell resolution in our previous study (Schubert et al., 2018), we were able to specifically assign certain projections to known clock neuronal morphologies and therefore to characterize the unique pattern of the DN_2_s. We found two main projections of the DN_2_s - one toward the medial brain passing to the contralateral hemisphere, the other projection running laterally. The latter runs dorsolaterally around the SLP and invades the anterior-lateral part of the AOTU. The loop around the SLP reminds strongly of the projections of the CRY-positive DN_1p_s described above. Since the staining with the *Clk206-GAL4;cry-GAL80* line was only partly satisfying, we looked for split-Gal4 lines that label specifically the DN_2_s.

**FIGURE 10:**
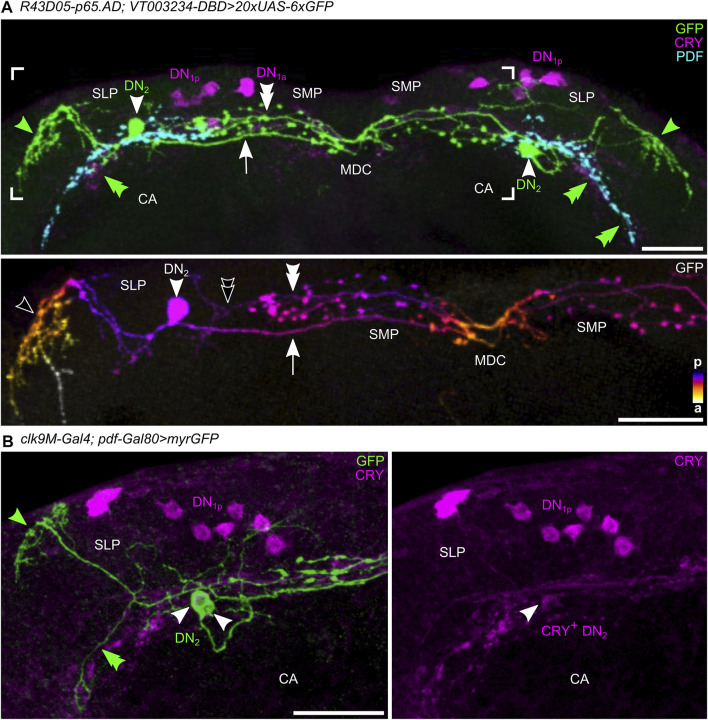
The morphology of the DN_2_s. **(A)** Maximal projection of 20 confocal planes showing a frontal view of the dorsal protocerebrum, in which one DN_2_ is marked with cytosolic GFP using the split-GAL4 line *R43D03-AD; VT003234* with the 20xUAS-6xGFP reporter) and counter-stained with anti-CRY (magenta) and anti-PDF (cyan). The lower panel shows the GFP labeling of the area marked above in greater detail in color-coded depth information. Fibers in the posterior (p) brain are marked blue and fibers further anterior (a) are marked in yellow/white. **(B)** Maximal projection of 26 confocal planes showing a frontal view of the left dorsolateral protocerebrum, in which two DN_2_s are marked with membrane-bound GFP (using *Clk9M-GAL4; Pdf-GAL80* with the *UAS-myrGFP* reporter) and counter-stained with anti-CRY (magenta). Note that one of the two DN_2_s is weakly CRY-positive (CRY^+^ DN_2_, right). Green arrowheads mark the DN_2_ fibers projecting to the anterior optic tubercle (AOTU), green double arrowheads mark fibers that follow the PDF terminals for several micrometers ventrally, green arrows point to the DN_2_ fiber running contralaterally, and thick green arrows to the varicose terminals on the contralateral side. From these, in some cases, weak, nonvaricose fibers continue toward the contralateral soma of DN_2_. MDC, middle dorsal commissure; CA calyx of the mushroom bodies. Scale bars represent 50 µm.

Indeed, the just described DN_2_ projection pattern was confirmed by using a split-GAL4 line that specifically labels one DN_2_ and none of the other clock neurons (*R43D03-AD; VT003234*; Sekiguchi et al., 2020, Supplementary Video S3) and by the *Clk9M-GAL4* line combined with *Pdf-GAL80*, which has been reported to label one or both DN_2_ (Kaneko et al., 2012; Goda et al., 2016). The new split-GAL4 DN_2_ driver was considerably weaker than the *Clk206-GAL4* and the *Clk9M-GAL4* driver, but we got satisfying labeling with a cytosolic GFP reporter that accumulates GFP stronger than the membrane-bound GFP does (Supplementary Table S1). In the case of *Clk9M-GAL4*; *Pdf-GAL80*, membrane-bound GFP was sufficient for labeling the DN_2_s. As expected, the novel split-GAL4 line marked only one DN_2_ and this cell was always CRY-negative (Figure 10A). With the *Clk9M-GAL4*; *Pdf-GAL80,* we could see two DN_2_s in only one brain (out of 16, Figure 10B). In all other brains, again only one DN_2_ was labeled, and in about half of the cases, the labeled DN_2_ was weakly CRY-positive (Figure 10B). This suggests that the two DN_2_s can be distinguished by their CRY expression level, and that *Clk9M-GAL4* drives GFP either in the CRY-negative or in a weakly CRY-positive DN_2_. Nevertheless, we did not see any difference in morphology between the different individually labeled cells, suggesting that the two DN_2_s are very similar. In the following, we will describe the typical morphology of the DN_2_s in more detail.

**FIGURE 11:**
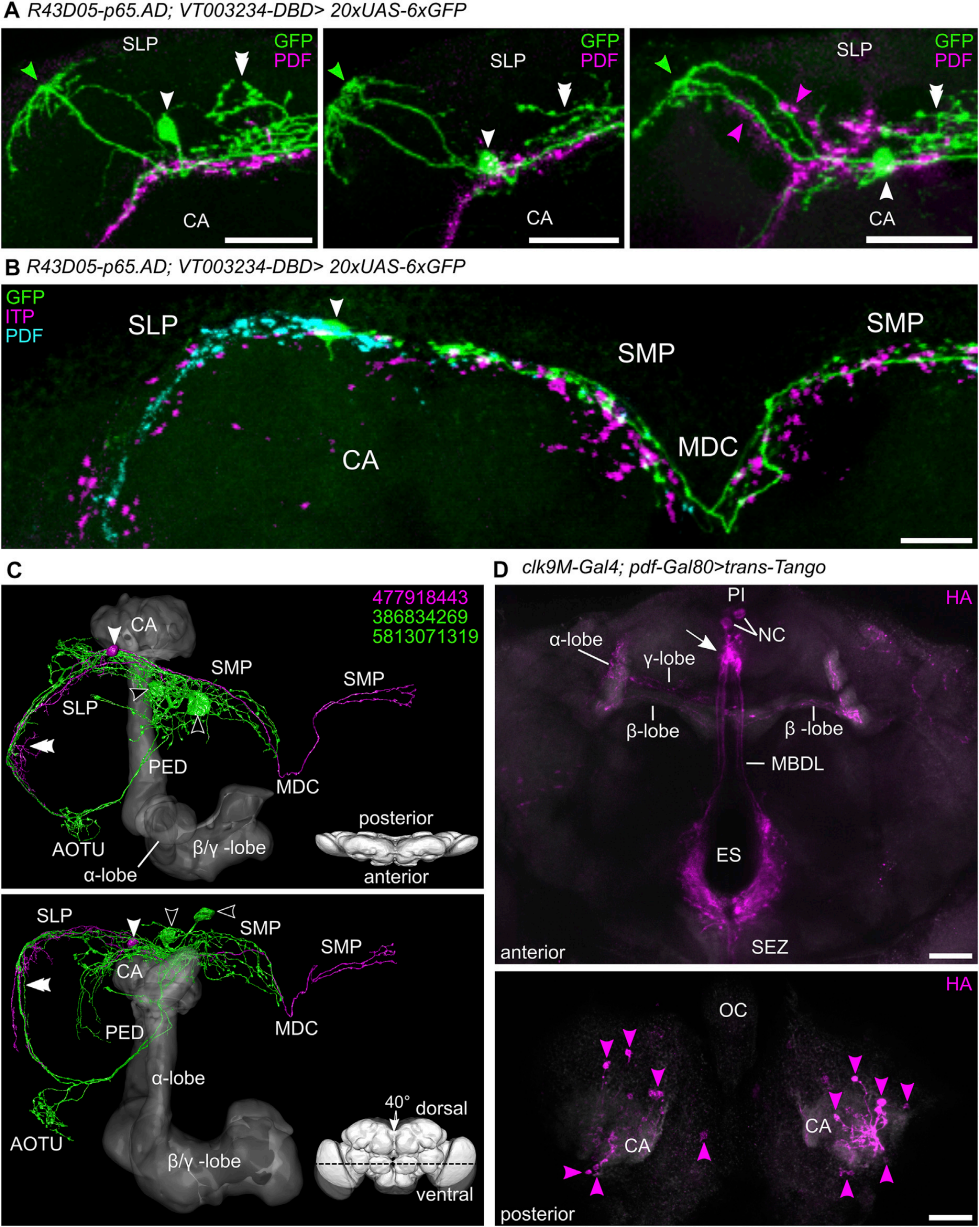
Vicinity of the DN2s to other clock neurons and putative downstream synaptic partners. **(A)** Confocal images (overlay of 27, 43, and 21 stacks, respectively) demonstrating the close vicinity of the DN_2_s (green) to the PDF-positive terminals of the s-LN_v_s (magenta). Labeling as in Figure 9. **(B)** Maximal projection of 18 confocal planes showing the close vicinity of the DN_2_s (green) to the ITP-positive LN_d_ and fifth LN (magenta) and the PDF-positive terminals of the s-LN_v_s (cyan). White arrows mark the overlap of ITP-positive fibers with the contralateral projecting fibers of the DN_2_s in the superior median protocerebrum (SMP) and the middle dorsal commissure (MDC). **(C)** Overlay between DN_2_ and CRY-positive DN_1p_ fibers running around the SLP to the anterior optic tubercle (AOTU). **(D)** Neurons postsynaptic to the DN_2_s revealed by *trans-*Tango. Top: maximal projection of eight confocal planes from the anterior brain depicting postsynaptic HA staining in neurosecretory cells (NC) running in the median bundle (MBDL) and around the esophagus (ES) to the subesophageal zone (SEZ) as well as in the α- and β-lobes of the mushroom bodies. The white arrow points to a dense HA-positive fiber net that lies exactly in the midline of the brain where the DN_2_s cross in the MDC (compare with **B**). Bottom: maximal projection of four confocal planes from the posterior brain showing postsynaptic staining in Kenyon cells (small magenta arrows) of the mushroom body as well as in the calyces (CA). OC, ocellar tract. Scale bars represent 25 µm.

**FIGURE 12:**
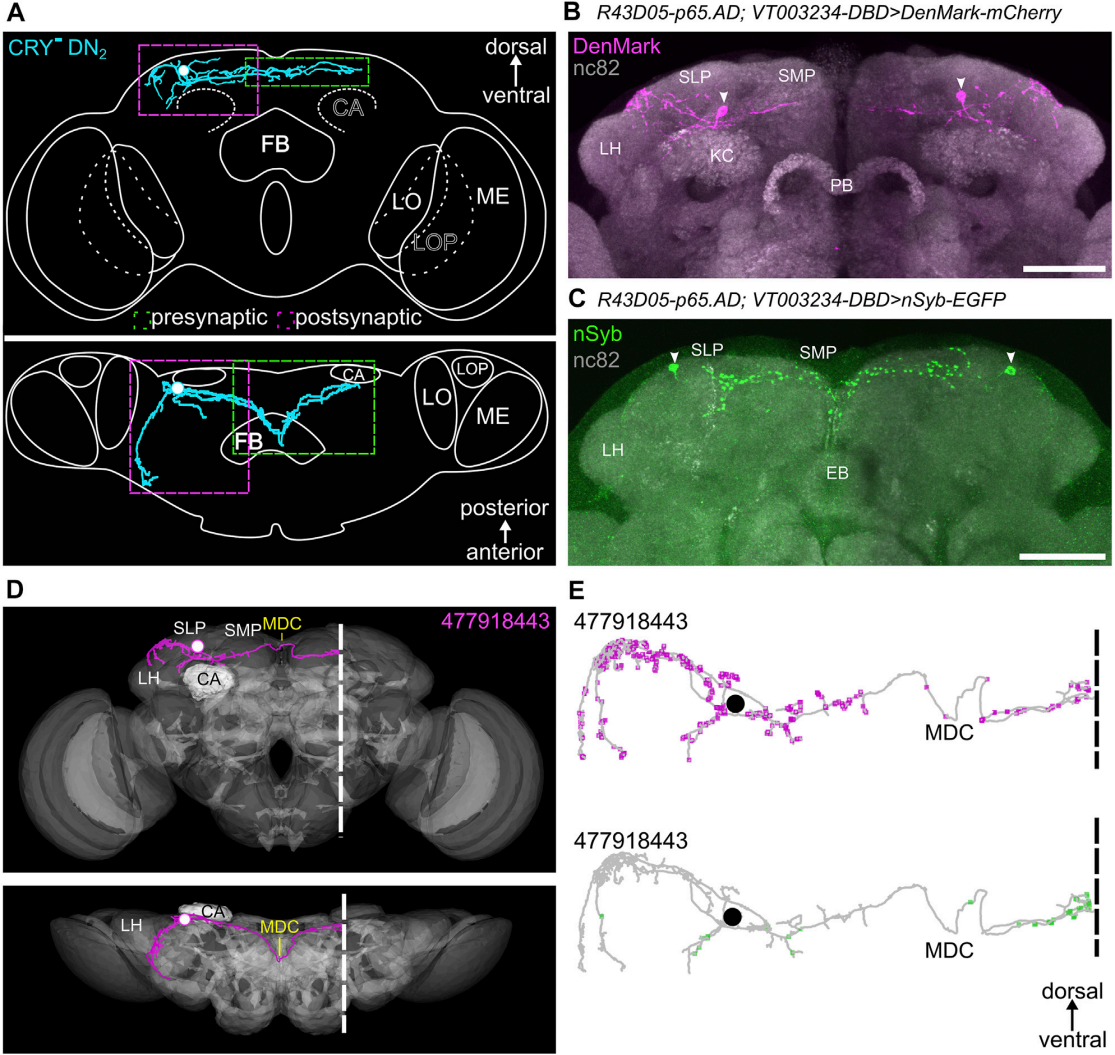
Morphology of DN_2_ #477918443 found in the hemibrain and its input and output sites. **(A)** Schematic summary of the morphology and the input (magenta) and output (green) sites of DN_2_ #477918443. **(B)** Staining against mCherry the fluorescent marker of DenMark which shows the dendritic sites of the DN_2_ addressed by the split-GAL4 line. While the dendritic signal is strong in the arborizations branching in the superior lateral protocerebrum (SLP) and the fibers running anterior at the border between the SLP and the lateral horn (LH), no or only weak signal could be recognized in the superior medial protocerebrum (SMP). **(C)** Staining against GFP to reveal the axonal endings marked by the enhanced GFP-(EGFP) tagged neuronal Synaptobrevin (nSyb). In contrast to the DenMark staining, the signal is mostly restricted to the SMP and only some or no signal could be recognized in the lateral brain. **(D)** Reconstruction of DN_2_ #477918443 on electron microscopic level found in the hemibrain. The DN_2_ shows only a few arborizations in the ipsilateral SMP but sends fibers through the middle dorsal commissure (MDC) to the contralateral SMP. Additionally, fibers are running and branching further on the ipsilateral border of SLP and LH anterior in the direction of the anterior optic tubercle (AOTU) without reaching it. The white dashed line shows the border of the hemibrain. **(E)** Postsynaptic (magenta, input) and presynaptic (green, output) sites of the DN_2_. The input sites of the DN_2_ are mainly restricted to the SLP as shown in the DenMark staining, while the output sites are largely absent since they are located in the contralateral SMP. ME, medulla; LO, lobula; LOP, lobula plate; FB, fan-shaped body; EB, ellipsoid body; CA, mushroom body calyx; PB, protocerebral bridge. Scale bars represent 50 μm.

Shortly after leaving the soma, the DN_2_ neurites bifurcate into two main branches. As found by Flybow, one runs to the contralateral brain hemisphere (arrow in Figure 10A), the other runs laterally (green arrowhead). The laterally running branch projects into the dorsal SLP and then runs further anteriorly forming the characteristic loop around the SLP toward the AOTU, but the DN_2_ fibers do not reach it (Figures 10–12). Often this branch splits into two prominent fibers (Figure 11A). Before the split-off of the lateral branch(es), some less intensively stained fibers turn ventrally and follow the PDF-positive s-LN_v_ terminals for several micrometers (Figure 10, green double arrowheads). Generally, the ipsilateral remaining DN_2_ fibers were in close vicinity to the s-LN_v_ terminals, suggesting an interaction between the two clock neuron types (*see* also below). In one case (out of 15 brains), fibers of the s-LN_v_ terminals even followed the dorsolaterally projecting DN_2_ fibers (magenta arrowhead in Figure 11A). The contralaterally projecting fibers of the DN_2_s showed pronounced varicosities and terminated in the superior protocerebrum slightly before they reached the contralateral DN_2_ somata (white double arrowheads in Figures 10A, 11A). Sometimes we saw fine non-varicose fibers extending from the varicose fibers further laterally (open double arrowhead, Figure 10A).

The arborizations of the DN_2_s did not only largely overlap with the PDF-positive fibers of the s-LN_v_s but also with those of the ITP-positive fibers from the fifth LN and LN_d_ (Figure 11B). This was especially true in the SMP to SLP, in which the arborizations of the ipsilateral DN_2_s were less varicose than those running to the contralateral hemisphere. As for ITP-positive clock neurons, the close vicinity to the DN_2_s was maintained throughout the SMP where both clock neuron types cross the midline of the brain in the MDC (Figure 11B).

#### Synapses and Putative Synaptic Partners of the DN_2_s

**FIGURE 13:**
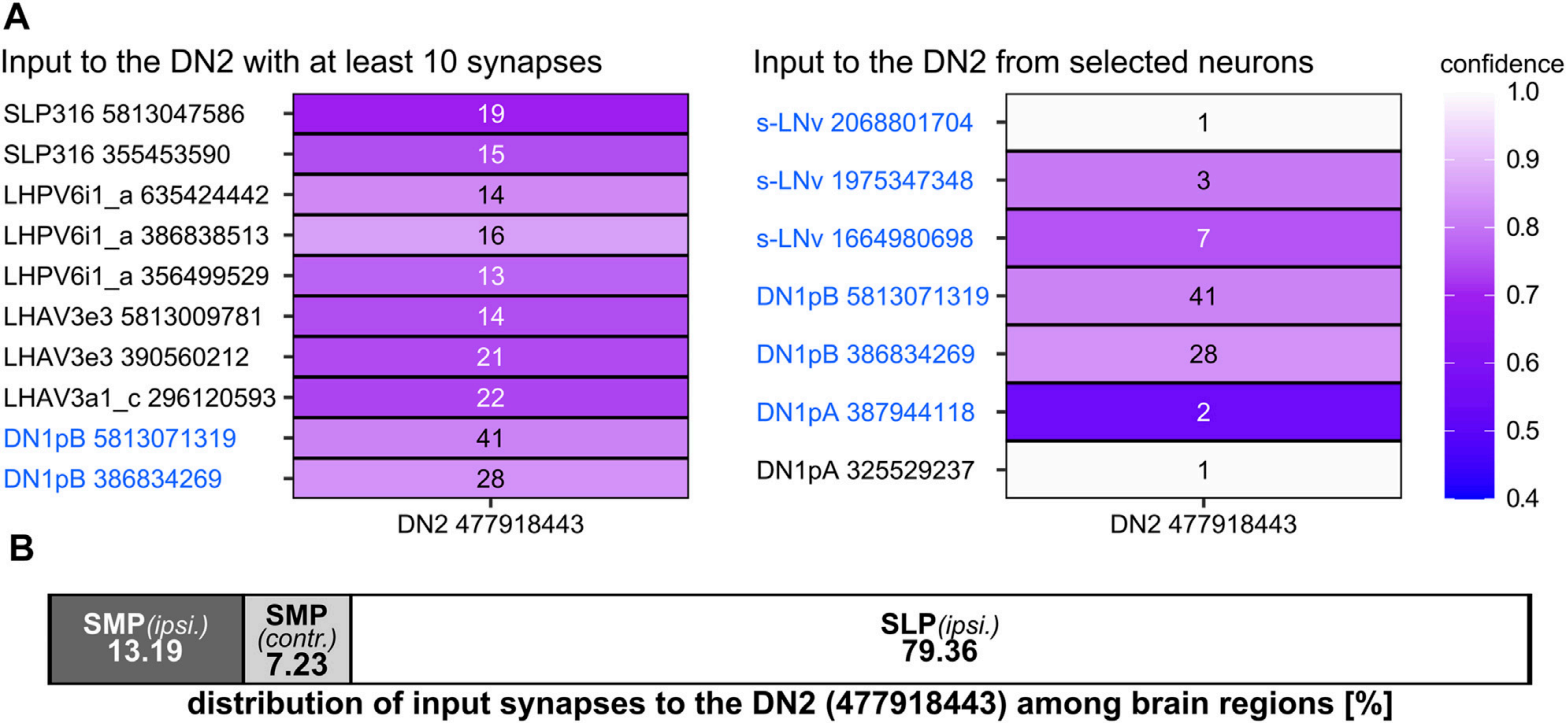
Presynaptic partners of DN_2_ #477918443 and the distribution of postsynaptic synapses among the brain regions. **(A)** Presynaptic partners of the DN_2_ with at least 10 synapses and selected clock neurons as presynaptic partners. The DN_2_ gets input from neurons with their cell bodies located in the superior lateral protocerebrum (SLP) as well as in the lateral horn (LH). Additionally, the DN_2_ gets input from the CRY-positive DN_1p_s as already visible in the postsynaptic partners of the CRY-positive DN_1p_s. Further, it gets weak input from some s-LN_v_s. **(B)** Distribution of the DN_2_ postsynaptic synapses (input) among the brain regions. The DN_2_ gets around 80% of the input in the ipsilateral SLP and only around 20% of the input in the ipsilateral (ipsi.) or contralateral (contr.) SMP.

So far, the neuromessengers used by the DN_2_s are unknown. The prominent varicosities in the contralaterally projecting terminals of the DN_2_s suggest modulatory neuropeptides that are released *via* volume transmission, but the DN_2_s may additionally utilize fast neurotransmitters operating *via* synapses. Single-cell transcriptome data (Ma et al., 2021) proposed glutamate as a neurotransmitter of the DN_2_s, but glutamate was not detected in the DN_2_s by immunohistochemistry (Hamasaka et al., 2007). Our polarity assessment by Synaptobrevin::EGFP and Denmark shows that the DN_2_s are strongly polarized (Figures 12B,C,E). They receive input in the ipsilateral brain hemisphere and have output synapses at the contralateral hemisphere. A very similar distribution of synapses was also found in neuron #477918443 of the hemibrain (Figures 12E, 13B). This neuron morphologically largely resembles the two DN_2_s (Figure 12D and Supplementary Video S3). As revealed by our polarity staining most input synapses are found on the DN_2_ fibers projecting anteriorly around the SLP toward the AOTU. These DN_2_ fibers largely intertwine with presynaptic fibers from the CRY-positive DN_1p_s (Figure 11C, double arrowhead), suggesting that the latter signal to the DN_2_s, an assumption that is strongly supported by the high number of output synapses from the CRY-positive DN_1p_s to the DN_2_s (Figures 9A, 13A). The only other clock neurons giving input to the DN_2_s are the s-LN_v_s (Figure 13A). This supports the findings of Tang *et al.* (2017) who could find putative synaptic contact between the s-LN_v_s and the DN_2_s using GFP Reconstitution Across Synaptic Partner (GRASP) (Tang et al., 2017).

*Trans-*Tango staining with the split-GAL4 and *GAL4*/*GAL80* DN_2_ lines turned out to be more equivocal than for the other clock neurons. This was the case because the *R43D03-AD; VT003234* split-GAL4 line was such a weak driver that we could not detect the DN_2_s in the presynaptic GFP channel, making it therefore difficult to trust the postsynaptic staining. The *Clk9M-GAL4; Pdf-GAL80* line, on the other hand, showed sometimes expression in the DN_1a_s that made us doubt that we can rely on all postsynaptic labeling we saw. Nevertheless, both drivers led consistently to HA-expression in Kenyon cells of the mushroom bodies and all their lobes (Figure 11D). Since the DN_1a_s do not signal onto the mushroom bodies and the DN_2_s neurites project close to the calyces of the mushroom bodies, it is quite likely that they indeed form synapses with specific Kenyon cells. In addition, in the stronger *Clk9M-GAL4; Pdf-GAL80* line, we found always postsynaptic labeling in the pars intercerebralis. In several brains, few neurosecretory cells of the pars intercerebralis were labeled, projecting through the median bundle down to the esophageal foramen and the subesophageal zone (Figure 11D). Since the neurites of the DN_2_s cross the midline of the brain at the exact location where we see dense HA labeling (arrow in Figure 11D), this staining pattern is likely true and the DN_2_s do indeed form synapses with neurosecretory cells of the pars intercerebralis.

It is also important to note that we did not see any postsynaptic staining in any of the clock neurons, suggesting that the DN_2_s might not form any output synapses with other clock neurons. In the hemibrain, no output synapses of the DN_2_ (#477918443) were annotated, because the latter are found on the contralateral hemisphere. Input to the DN_2_s came mainly from the ipsilateral SLP and here mainly from the CRY-positive DN_1p_s (Figure 13).

### The DN_3_s

**FIGURE 14:**
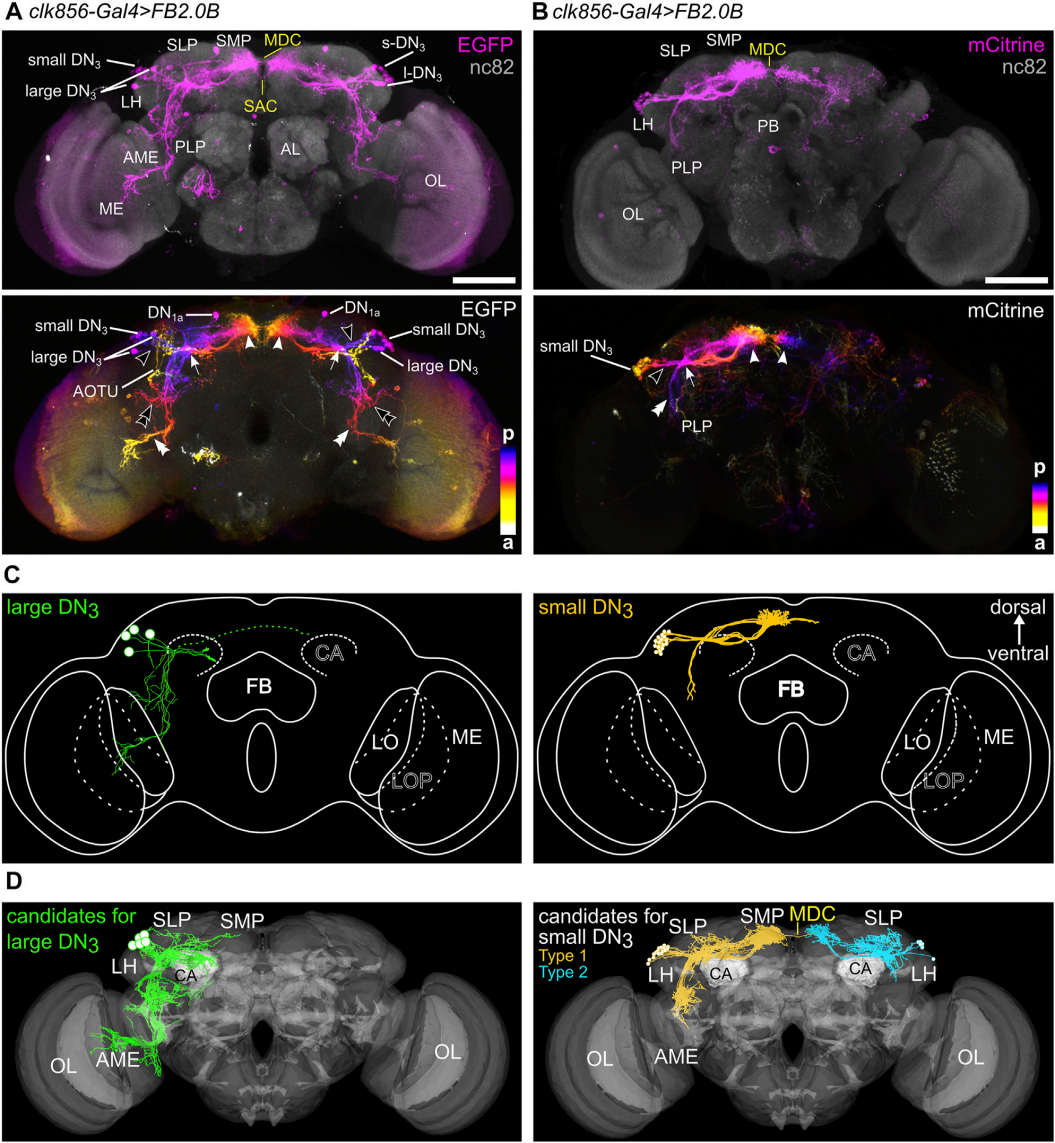
The morphology of the DN_3_ subclasses. **(A**,**B)** Two exemplary brains, which express the Flybow-reporters in different subsets of DN_3_ (maximal projections of 57 **(A)** and 32 **(B)** confocal planes). The upper panel shows an overlay with neuropil counterstaining (nc82), the lower panel shows the neurons with color-coded depth information. Fibers in the posterior (p) brain are marked blue and fibers further anterior (a) are marked in yellow/white. **(A)** Fibers in the accessory medulla (AME) could only be observed in brains, in which the larger DN_3_s were labeled. Likewise, the projections through the superior arch commissure (SAC) are absent in brains, in which the large DN_3_s are not labeled **(B)**. **(B)** Arborization pattern of the small DN_3_s. The projections of these cells in the posterior lateral protocerebrum (PLP) do not invade the AME. The fibers run through the middle dorsal commissure (MDC), but not *via* the SAC into the contralateral hemisphere. **(C)** Estimated projection pattern of the large and small DN_3_s. All DN_3_s have arborizations in the dorsal brain, but the contribution to the SAC is unique to the larger cells. The overlap with projections from other neurons (mainly small DN_3_s) made further analysis of the DN_3_ arborization pattern in the dorsal brain impossible (indicated by dashed line). **(D)** Reconstruction of potential DN_3_ candidates from the hemibrain. According to their morphology, the putative DN_3_s can be divided into three subtypes (green, yellow, cyan). While the green neurons look similar to the above-described morphology of the large DN_3_s the yellow neurons resemble the described morphology of the small DN_3_s (compare with **C**). The cyan depicted neurons are not matching the described morphologies based on the Flybow staining. They possess the arborization pattern of the small DN_3_s in the superior lateral protocerebrum (SLP) but lack the fibers projecting ventrally to the posterior lateral protocerebrum (PLP). These neurons could be a further subtype of the DN_3_s, which was not detected by the Flybow staining. Candidates for the large DN_3_: #510602264, #5813021221, #356140100, #5813057153, #5813020750; candidates for (DN_3_) small DN_3_ type 1: #357949102, #5813010377, #358285424, #358976515, #450371612, #478937243, #510680957, #571364144, #664447502, #5813026780, #5813032640, #451424866, #574062996 (DN_3__A). Candidates for small DN_3_ type 2: #326465302, #356835277, #386847799, #417536905, #417882515, #479260921, #358631450, #328273806 (DN_3__B). LH, lateral horn; ME, medulla; LO, lobula; LOP, lobula plate; FB, fan-shaped body; EB, ellipsoid body; CA, mushroom body calyx; PB, protocerebral bridge; AL, antennal lobe. Scale bars represent 50 μm.

Comprising approximately 40 cells, the DN_3_s are by far the largest clock neuron cluster in the brain of the adult fly. Several studies have already revealed the presence of larger and smaller DN_3_ cell bodies, and that some of them are situated more anteriorly in the brain compared to the others (Helfrich-Förster, 2003; Shafer et al., 2006; Helfrich-Förster et al., 2007b). This is in line with our observations made on 43 brains, in which the Flybow-reporters were expressed under the control of the pan-clock-neuronal *Clk856-GAL4* driver. The somata of approximately 15 DN_3_s are located posterior to the LH, whereas the remaining cells reside more anteriorly in the dorsal and lateral cell body rind of the LH, respectively. The majority of DN_3_s possess rather small cell bodies and only about four to five neurons have larger somata. Half of the larger cells are found among the small posterior DN_3_s, while the other two are found among the more anteriorly located neurons of this group. Interestingly, the projections of the large DN_3_s strongly resemble the arborization pattern of the ITP- and CRY-positive LNs (fifth LN and LN_d_) (Schubert et al., 2018). Their projections initially run along the posterior boundary between the LH and the SLP (Figures 14A,B open arrowhead). The first branching occurred on the posterior surface of the brain at the level of the trijunction between the LH, SLP, and SCL (Figures 14A,B, arrows). Here, some of the fibers from the large DN_3_ projected anteriorly to the AOTU *via* three paths. The fiber bundle running along the first path, projects around the surface of the SLP. This is the same path (or better loop) used by the CRY-positive DN_1p_s and the DN_2_s projecting towards the AOTU. The second path towards the AOTU coincides with the second loop of the CRY-positive DN_1p_s that runs along the trijunction of SLP, SCL, and superior intermediate protocerebrum. The third path originates in the lateral PLP and runs along the surface of the anterior ventrolateral protocerebrum. These different fiber bundles running in the different paths are hard to identify in Figure 14 (anterior projecting fibers in yellow) and are best seen in Supplementary Video S4.

Other fibers of the DN_3_s remained initially posterior (blue colors in Figure 14A) and passed ventrally through the PLP until they reached the ipsilateral AME (Figure 14A white double arrowhead). On their way to the AME, numerous side-branches separated from the main bundle and formed a kind of fiber hub (Figure 14A double open arrowhead) where they overlap with the ITP- and CRY-positive LNs (*see* Supplementary Video S5).

Most fibers of the DN_3_ projected medially forming a dense varicose fiber network in the SMP (Figures 14A,B, arrowheads). From there, a portion of the projections crossed the midline of the brain *via* the MDC and superior arch commissures (SAC) reaching into the contralateral hemisphere (Figures 14A,B).

We observed a different arborization pattern in brains in which only the small DN_3_s were labeled compared to brains in which both, the large and small DN_3_s were stained (Figure 14B). In these brains, the two hemispheres were connected exclusively *via* the MDC, with no additional projections through the SAC (Figure 14B). However, there are even more apparent differences between the small and large DN_3_s: the small DN_3_s lack the fiber bundle towards the AOTU and the projections ending in the AME. The fibers of the small DN_3_s, that pass through the PLP toward the AME terminate in the fiber hub of the PLP halfway to the AME described above (Figure 14B double arrowheads). Thus, only the four to five large DN_3_s send fibers into the AOTU and AME (Figure 14C).

No DN_3_s could be annotated with certainty in the hemibrain dataset until now. However, there are possible candidates available of which three subtypes can be distinguished. While two subtypes comprise only five (named DN_3_) and six (named DN_3_B) neurons, respectively, the third subtype comprises 15 neurons (named DN_3_A) (Supplementary Video S4). Comparing the morphology of these DN_3_ candidates with our findings, we are fairly confident that the subtype with five neurons comprises the large DN_3_s, as their projection pattern is consistent with our descriptions (Figure 14D). The only difference is that there are no fibers reported running through the SAC in the hemibrain that we observed in our Flybow staining, but this might be caused by a general lack of contralateral projections in the hemibrain, which we discussed previously (Reinhard et al., 2022).

The second group of DN_3_s candidates from the hemibrain that matches our description is the DN_3_A subtype comprising 15 neurons. These neurons largely resemble the small DN_3_s. As we found in our staining they project halfway down to the AME and terminate in the PLP fiber hub.

The third subtype of DN_3_ candidates in the hemibrain completely lacks the projections extending ventrally towards the AME in the PLP. Otherwise, it looks similar to the small DN_3_s. We did not see this subtype in our staining, but most likely, the DN_3_s comprise more subtypes than we and others could identify so far.

The small DN_3_s were also labeled by Guo *et al.* (2018) as common neurons between the *Clk856-GAL4* and the *R77H08-LexA* driver lines (Guo et al., 2018 Supplementary Figure S1A). Furthermore, Meiselman *et al.* (2022) recently found the large (and several small DN_3_s) labeled by a specific split-GAL4 line. These authors report the same arborization pattern that we describe here. They also found a faint commissure in the superior brain, but unfortunately, we could not distinguish between the SAC and MDC according to their figures (Figure 2K in Meiselman et al., 2022).

Due to the lack of specific driver lines, we have not yet been able to perform a polarity analysis of the DN_3_s. It will be most promising to do so in the future with the recently published split-GAL4 line. In addition, we assume that the hemibrain DN_3_ candidates will be certainly annotated as DN_3_s as soon as further experiments allow a more accurate comparison in respect of their polarity and synaptic partners.

## Discussion

This study aimed to unravel the morphology and connectivity of *Drosophila*’s dorsal clock neurons. Throughout the results, we have compared our findings with those of previous studies, which we largely confirm. Nevertheless, we found new details that reveal a so far unknown degree of heterogeneity within certain clock neuron groups that might impact their function. Only the two DN_1a_s and two DN_2_s appear to have similar morphology. This observation is supported by single-cell RNA sequencing analysis that found no qualitative differences within the neurons of these groups (Ma et al., 2021). At the same time, the DN_1a_s and DN_2_s (together with the four s-LN_v_s and the fifth LN) are functional from the first larval instar onward making them basic clock neurons that might be of particular functional importance (Kaneko et al., 1997). In the following, we will discuss our findings in the light of numerous functional studies that have attributed specific roles to the clock neuron groups, with a special emphasis on the DN_1a_s and DN_2_s.

### Putative Functional Impact of the DN_1a_s

The DN_1a_s express two neuropeptides (CCHamide1 and IPNamide) (Shafer et al., 2006; Fujiwara et al., 2018) and one neurotransmitter (glutamate) (Hamasaka et al., 2007). They are functionally strongly interconnected with the s-LN_v_s *via* neuropeptide signaling. While the s-LN_v_s signal to the DN_1a_s *via* PDF (Shafer et al., 2008; Im et al., 2011), the DN_1a_s signal to the s-LN_v_s *via* CCHamide1 (Fujiwara et al., 2018). Here, we show by *trans-*Tango that the two neurons are additionally interconnected *via* regular synapses. This fits with previous *trans-*Tango studies (Song et al., 2021) as well as immunohistochemical results demonstrating that the s-LN_v_s express metabotropic glutamate receptors that can be activated by glutamate from the DN_1a_s (Hamasaka et al., 2007). *Vice versa*, the DN_1a_s might express glycine receptors that can be activated by glycine released from the s-LN_v_s, although this has not yet been proven (Frenkel et al., 2017). Manipulations of the reciprocal peptidergic connections between the DN_1a_s and the s-LN_v_s affect the flies’ free-running period, their morning and evening activity as well as their siesta under light-dark conditions (Fujiwara et al., 2018). The synaptic connections between the two neuron groups have not been specifically manipulated, but the knockdown of glutamate receptors in the s-LN_v_s increases the free-running period and the nocturnal activity under light-dark conditions (Hamasaka et al., 2007). Furthermore, downregulation of glutamate synthetase in all dorsal clock neurons increases the rhythmicity of flies under constant light conditions (de Azevedo et al., 2020), indicating that glutamate signaling from the DNs is involved in the general light sensitivity of the clock. Although it is unlikely that this effect is mediated exclusively by the DN_1a_s (some DN_1p_s and DN_3_s are also glutamatergic as shown by Hamasaka *et al.* (2007) and Collins *et al.* (2012)), the DN_1a_s appear to contribute strongly to the light detection. At night, their silencing reduces light-induced startle responses, whereas during the day, PDFR-dependent signaling to the DN_1a_s limits light-induced startle responses (Song et al., 2021). As we show here, the DN_1a_s get strong input from the dorsal and lateral accessory calyces. The dorsal accessory calyces transfer visual information from the lobula (Li et al., 2020). Thus, DN_1a_s may directly receive light information from the optic lobe.

On the other hand, the lateral accessory calyces convey thermosensory information from neurons in the antennal lobes (Marin et al., 2020). While about half of the clock neurons are intrinsically light-sensitive (*via* CRY), none of them is per se temperature-sensitive (Sehadova et al., 2009) but they rely on temperature input from peripheral sensory neurons (the aristae on the antennae, the chordotonal organs and the antennal AC neurons (Tang et al., 2017; reviewed in; George and Stanewsky, 2021)). Indeed, the DN_1a_s get strong synaptic input from several temperature sensing projection neurons from the antennal lobes. Alpert *et al.* (2020) showed that temperatures below the preferred temperature for flies (∼25°C) silence the DN_1a_s *via* GABAergic synapses from such thermosensory projection neurons. This silencing leads to reduced activity and increased sleep in the morning and evening, which can only be overwritten by light and PDF signaling. Indeed, we found that the DN_1a_s signal not only to the morning neurons (the s-LN_v_s) but even stronger to the evening neurons, namely the CRY- and ITP-positive fifth LN and LN_d_. In addition, the DN_1a_s have few output synapses to tangential neurons arborizing in the second and eighth layer of the dFB, which overlap with the fibers of several clock neurons in the PLP. The dFB is known to be involved in the control of sleep (reviewed in Bertolini and Helfrich-Förster, 2021).

Altogether these results demonstrate that the DN_1a_s play a central role in light- and temperature integration of the circadian clock and can prominently influence the morning and evening neurons as well as the sleep of flies.

### Putative Functional Impact of the DN_1p_s

We could show that the DN_1p_s consist of more than the originally described two types of CRY-positive and CRY-negative neurons. In the hemibrain, seven of the ∼15 DN_1p_s are annotated. The seven neurons are classified into type A and type B neurons, but the type A neurons appear heterogeneous. At least one type A neuron looks different from the others (green neuron in Figure 6). We found the same neuron type by Flybow and identified an additional one that was not yet annotated in the hemibrain. Lamaze *et al.* (2018) depict a further DN_1p_ type in their supplement that was neither identified in the hemibrain nor in the present study. These two previously undescribed DN_1p_s project contralaterally like the type A neurons but differ in their projections toward the AOTU and PLP. The DN_1p_ identified here lacks the anterior projections to the AOTU but shows prominent fibers to the PLP (Figure 6D right panel), whereas the DN_1p_ identified by Lamaze projects anteriorly to the AOTU and lacks fibers in the PLP (Supplementary Figure S1A in Lamaze and Stanewsky, 2020). These findings demonstrate that the DN_1p_s encompass many neurons with different morphology and most likely also different neurochemistry. Single-cell RNA sequencing data underline this conclusion: according to their gene expression, the DN_1p_s comprise five to six distinct clusters (Ma et al., 2021). This heterogeneity could explain the different and partly contradicting findings regarding their function(s) (reviewed in Lamaze and Stanewsky, 2020). We will not repeat these details here but instead, focus on the main findings adding new insights that derive from the present study.

The DN_1p_s appear to comprise morning and evening neurons (Chatterjee et al., 2018), which is strongly supported by our findings. Type A DN_1p_s receive strong synaptic input from the evening neurons (ITP positive fifth LN and LN_d_) and signal even stronger back to them. Thus, there exists a prominent mutual connection between the type A DN_1p_s and the evening neurons, suggesting that the five annotated type A DN_1p_s are by themselves evening neurons. Nevertheless, the seven neurons are heterogeneous and at least one of them (green neuron in Figures 5, 6) gets synaptic input in the PLP, a region where many clock neurons arborize. This DN_1p_ may additionally receive input from morning neurons (s-LN_v_s) *via* PDF or from the LPNs *via* DH31 (Reinhard et al., 2022). So far this is the only annotated type A DN_1p_ neuron that is most likely CRY-positive, expresses the PDF receptor and additionally receives synaptic input in the PLP. It is tempting to speculate that all DN_1p_s that overlap with the s-LN_v_ projections in the PLP are CRY-positive, but this has to be proven by future studies. The type B DN_1p_s are all CRY-positive, largely overlap with projections from the s-LN_v_s, and express the PDFR. Thus, they are connected *via* PDF with the morning neurons and can contribute to the morning activity of the flies.

At the same time, DN_1p_s consist of sleep- and activity-promoting neurons (Guo et al., 2018; Lamaze et al., 2018). Through their fibers projecting to the pars intercerebralis, they affect wakefulness by signaling to neurosecretory cells expressing the *Drosophila* homolog of the mammalian corticotropin-releasing factor (Cavanaugh et al., 2014; Kunst et al., 2014). In contrast, the anterior projections of the DN_1p_s to the AOTU, appear to play a prominent role in sleep control because in the AOTU, CRY-positive type B DN_1p_s signal on sleep-promoting ring neurons of the ellipsoid body, influencing their neuronal activity (Lamaze et al., 2018). Here, we could confirm the intimate connection of the DN_1p_s to the AOTU. Moreover, we could show that the projections of the DN_1p_s toward the AOTU largely overlap with fibers from the DN_2_s and large DN_3_s. The DN_1p_s strongly signal *via* these projections to the DN_2_s (*see* below).

DN_1p_s are also known to promote the siesta of the flies (Guo et al., 2016) and to inhibit activity at low temperatures (Yadlapalli et al., 2018). They respond to cool temperatures (∼16°C) with a strong increase in firing rate, while they are inactive at warm temperatures (>25°C) (Yadlapalli et al., 2018). In contrast to the DN_1a_s, the DN_1p_s do not arborize in the lateral accessory lobes, but they seem to receive temperature information from thermoreceptors in the aristae of the antennae and chordotonal organs in the body *via* signals from anterior cells (AC) which are known to be involved in temperature sensing pathways (Hamada et al., 2008; Yadlapalli et al., 2018; Jin et al., 2021). Thus, the DN_1p_s appear to be a major gateway for temperature sensing into the circadian neural network, which continuously integrates temperature changes to coordinate the timing of sleep and activity. Most likely, the DN_1p_s pass this information to other clock neurons, which do not receive temperature input directly but are similarly involved in temperature-related behavior (e.g. DN_2_, Kaneko et al., 2012; Goda et al., 2016).

It is worth noting that the so far annotated type A DN_1p_s are quite polar and have their synaptic output sites in the contralateral SMP. The same is true for the DN_2_s, which we will discuss in the following. This feature might be important for integrating information from both hemispheres.

### Putative Functional Impact of the DN_2_s

The two DN_2_s, similarly to the DN_1a_s, show a special association with the s-LN_v_s. All three clock neuron groups express PER cyclically already during the larval stages, whereas PER in the larval DN_2_s cycles in antiphase to the other clock neurons (Kaneko et al., 1997). This anti-phase cycling depends on PDF signaling from the s-LN_v_s to the DN_2_s (Picot et al., 2009) suggesting that the DN_2_s are PDFR positive, although they are reported to be CRY-negative in adults (Benito et al., 2008; Yoshii et al., 2008). Here, we report weak CRY-immunostaining in one of the DN_2_s, strongly suggesting that the PDFR is also present since they are always co-expressed (Im et al., 2011).

The DN_2_s are important for temperature entrainment in larvae (Picot et al., 2009) and temperature preference in adults (Kaneko et al., 2012). In contrast to the DN_1a_s, the DN_2_s do not arborize in the lateral accessory calyces and we could not detect any direct input from temperature sensing neurons of the antennal lobes. Nevertheless, the DN_2_s could get information indirectly *via* other clock neurons. The most promising candidates are the type B CRY-positive DN_1p_s that get input from thermosensory AC neurons and that strongly contact the DN_2_s. Furthermore, Tang *et al.* (2017) suggested that AC neurons project also directly to the DN_2_s. In addition to synaptic input, the DN_2_s may receive neuropeptidergic input from the DN_1p_s *via* DH31 (Goda et al., 2016).

The DN_2_s themselves do barely signal to other clock neurons, suggesting that they are pure output neurons of the clock network. As mentioned, they are highly polar as they receive synaptic input in the ipsilateral SLP and have output synapses in the contralateral SMP. As we show here by *trans-*Tango, their main output sites are the mushroom bodies and neurosecretory cells in the pars intercerebralis. These two sites are highly involved in the regulation of sleep, arousal, and metabolism (*see* above and discussion in Reinhard *et al.* (2022)). Consequently, DN_2_s can impact both processes and adapt them to different environmental temperatures.

### Putative Functional Impact of the DN_3_s

The DN_3_s are the largest and, until very recently, also the less well-characterized group of the dorsal neurons. They are heterogeneous, not only regarding their size and morphology but also in respect to their gene expression, which divides them into two clusters (Ma et al., 2021). Here, we confirm previous findings that the DN_3_s consist of neurons with large and small somata with different morphologies, but they may be composed of further subgroups, as suggested by data from the hemibrain.

Some (∼4) of the DN_3_s express a homolog of the vertebrate circadian gene *nocturnin*, coding for a deadenylase involved in mRNA decay (Nagoshi et al., 2010), and up to 17 DN_3_s express the gene *quasimodo*, coding for a Zona pellucida domain protein (Chen et al., 2011; Buhl et al., 2016). Both proteins are involved in the light perception of the clock. Other observations suggest an additional role of the DN_3_s in the sensory integration of temperature cues into the circadian system (Harper et al., 2016). Consistent with this observation, up to 10 DN_3_s neurons express the DH31 receptor, and together with the DN_2_s cells, they appear necessary for regulating the temperature preference of the flies at the beginning of the night (Goda et al., 2016). The large DN_3_s are additionally responsive to PDF (Shafer et al., 2008). Furthermore, some DN_3_s express glutamate, and the majority of them express the neuropeptide AstC (Guo et al., 2018; Díaz et al., 2019; Meiselman et al., 2022). Díaz et al. (2019) suggest that the AstC-positive DN_3_s signal to at least one LN_d_ and in this way influence the evening activity of the flies. Guo *et al.* (2018) demonstrate a sleep-promoting role of the DN_3_s, especially of the small DN_3_s. Meiselman *et al.* (2022) show that the AstC-positive DN_3_s regulate the cold-induced dormancy of the flies, supporting the role of the DN_3_s in temperature-dependent behavior.

All these different roles are supported by morphological data. Especially the arborizations of the large DN_3_s reach the entire clock network. They form arborizations in all four fiber hubs of the clock network (AME, SMP, ventral PLP and the loop in the SLP). In the AME they might not only receive light input (Li et al., 2018) but also communicate with all lateral neurons. In the SMP, they overlap with all clock neurons besides the LN_v_s. In the ventral PLP hub (double open arrowhead in Figure 14A) their fibers largely overlap with the cell bodies of the LPNs and with fibers from the evening neurons (ITP-positive fifth LN and LN_d_) and the small DN_3_s. In the SLP loop, they are again in close contact with the evening neurons as well as with many DNs. Consequently, they may communicate with all these neurons. From our *trans*-Tango data, we already know that one to two larger DN_3_s get input from the DN_1a_s. Connectivity data from the hemibrain suggest weak synaptic input from the type B CRY-positive DN_1p_s to some of the small DN_3_s. The large DN_3_ even appear to have dense arborizations in the lateral accessory calyces, which would predispose them to receive information from the thermosensory neurons of the antennal lobes. Moreover, they cross the midline of the brain in two commissures, meaning that they can integrate information from both brain hemispheres. The small DN_3_s form a prominent fiber network in the SLP and SMP and run ventrally in the PLP where most of them appear to terminate in the fiber hub formed by the arborizations of the large DN_3_s, fifth LN, and LN_d_. Thus, they contribute to the wide connectivity of the large DN_3_s within the clock network.

## Conclusion

**FIGURE 15:**
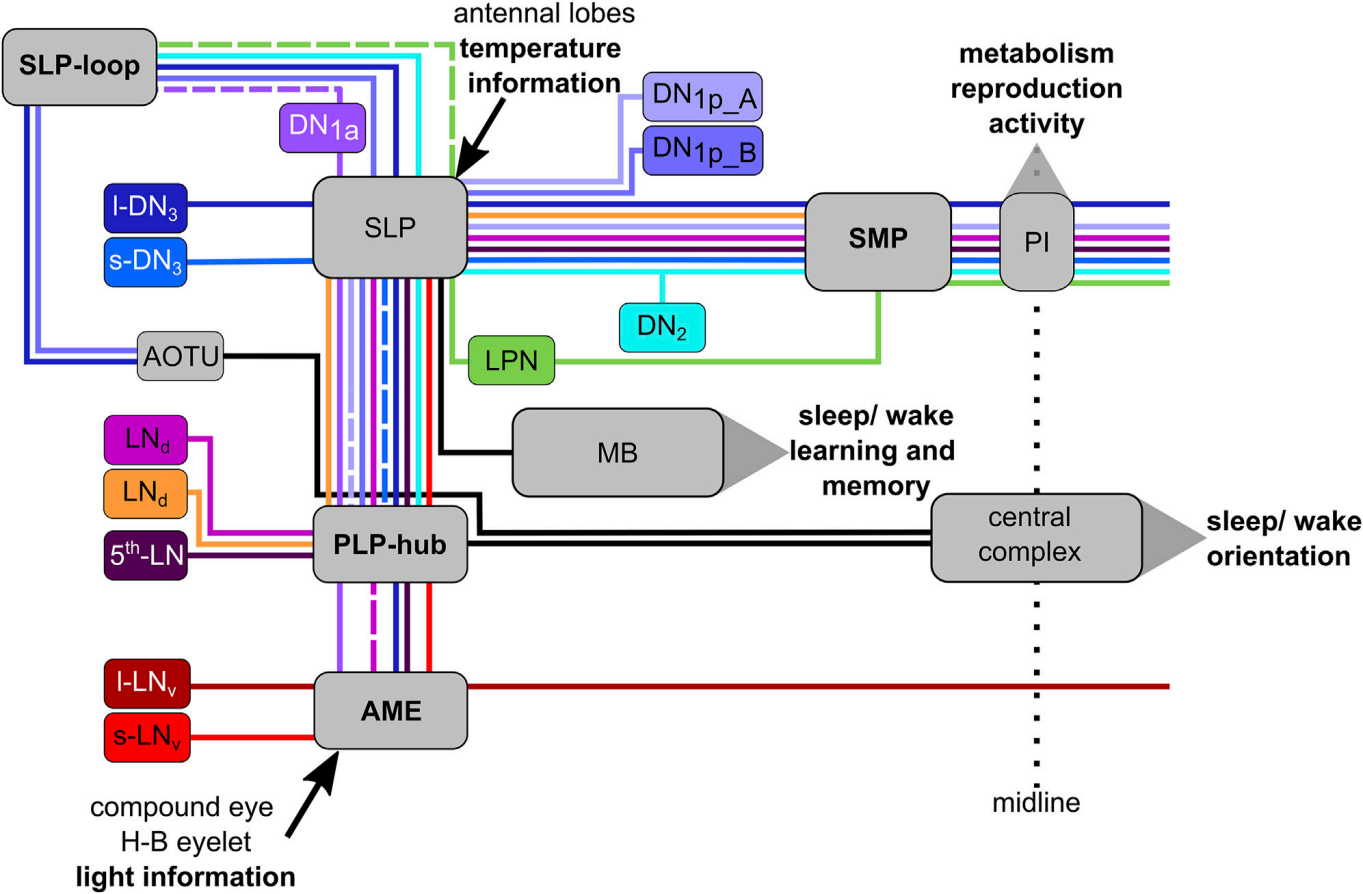
Schematic overview of the *Drosophila* clock network in the left hemisphere including input and output sites. The major groups of clock neurons and their corresponding projections are depicted in color, while the brain regions they invade are shown in grey. Bold letters highlight major hubs and tracts, in which the fibers of several clock neurons intermingle and contact each other. These are the “accessory medulla” (AME), the newly discovered “posterior lateral protocerebrum hub” (PLP-hub), the “superior lateral protocerebrum loop” (SLP-loop) and the “superior medial protocerebrum” (SMP). Dashed colored lines indicate that not all neurons of the relevant group show the drawn projections. Besides the intrinsical light-sensitivity of some clock neurons (*via* CRY), the clock network receives light input from the compound eyes and the Hofbauer-Buchner (H-B) eyelets in the AME, while it receives temperature information solely from peripheral sensory organs in the chordotonal organs antennae and antennal lobes in the “superior lateral protocerebrum” (SLP). Output from the clock network to control rhythms in metabolism, reproduction and activity occurs in the “pars intercerebralis” (PI), while the rhythmic control of the sleep/wake cycle occurs *via* connections to the mushroom bodies (MB) and the central complex. The MBs fulfill additional functions such as learning and memory that are also modulated by the clock network. The central complex consists of the protocerebral bridge, the fan-shaped body, the ellipsoid body and the noduli and has additional important roles in orientation and navigation. Flying straight for several hours needs the input from the clock to compensate for the movements of the sun across the sky. This input may come *via* the DN_1p__B (also called anteriorly projecting DN_1p_) and the DN_3_ with large somata (l-DN_3_) that run to the anterior optic tubercle (AOTU). The anterior tubercle neurons are connected with the ring neurons of the central complex of which some are involved in orientation and others in sleep. Certain neurons of the fan-shaped body have synaptic contact with clock neurons in the PLP hub and by this way may carry rhythmic information into the central complex.

Here, we show that the DNs are an integral part of the clock network. Functionally, they appear to integrate light and temperature information into the clock network and contribute to the control of many behavioral outputs reaching from sleep and activity to the control of metabolism *via* the neurosecretory system of the fly (sumarized in Figure 15). Anatomically, we show that the fibers of the DNs intermingle with those of the LNs (Supplementary Video S5). Besides the already known AME and the SMP (often referred to simply as the dorsal protocerebrum), we identify two areas in the brain, in which the interplay between all clock neurons appears especially intimate. The first area is the SLP, where several clock neurons join a fiber tract, which forms a loop around the SLP and terminates in the AOTU. These are the CRY-positive type B DN_1p_s, the DN_2_s, the large DN_3_s, the LPNs, and the ITP- and CRY-positive fifth LN and LN_d_. The second area lies in the PLP very close to the cell bodies of the LPNs, where the s-LN_v_s pass through and the large and small DN_3_s, the DN_1a_s, and the ITP- and CRY-positive fifth LN and LN_d_ contribute to a fiber hub. These two brain areas may serve as additional communications centers for the clock neurons. They may also serve as additional input and output centers of the clock. Here, we show that the clock network signals to certain dFB neurons *via* the PLP hub and Kind *et al.* (2021) have recently demonstrated connections from the visual system to the AME and the PLP hub as well as the AOTU close to the loop in the SLP. It will be most promising to unravel these connections in further detail.

## Data Availability

The raw data supporting the conclusions of this article will be made available by the authors, without undue reservation.

## Glossary

AME: accessory medulla
AOTU: anterior optic tubercle
AstC: Allatostatin C
CA: mushroom body calyx
*contr.*: contralateral
CRY: Cryptochrome
DenMark: dendritic marker
dFB: dorsal fan-shaped body
DH31: diuretic hormone 31
DN: dorsal neurons
**DN**_**1a**_: anterior dorsal neurons group 1
**DN**_**1p**_: posterior dorsal neurons group 1
**DN**_**1p**_ **_A**: posterior remaining subtype of DN_1p_ (hemibrain nomenclature), also known as vc-DN_1p_ ()
**DN**_**1p**_ **_B**: anterior projecting subtype of DN_1p_ (hemibrain nomenclature), also known as a-DN_1p_ ()
**DN**_**2**_: dorsal neurons group 2
**DN**_**3**_: dorsal neurons group 3
EGFP: enhanced green fluorescent protein
FAFB: full adult female brain (electron microscopic dataset)
FB: fan-shaped body of the central complex
FB2.0B: *UAS-Flybow2.0B*; refers to the Flybow system
GFP: green fluorescent protein
HA: hemagglutinin
hs-Flp: heat shock FLP recombinase
ICL: inferior clamp
*ipsi.*: ipsilateral
ITP: ion transport peptide
LD: light-dark
**l-DN**_**3**_: large dorsal neurons 3
LH: lateral horn
**l-LN**_**v**_: large ventrolateral neurons
**5**_**th**_ **LN**: 5^th^ lateral neuron
LN: lateral neurons
**LN**_**d**_: dorsolateral neurons
LO: lobula
LPN: lateral posterior neurons
MDC: middle dorsal commissure
ME: medulla
myrGFP: myristoylated GFP
nSyb: neuronal Synaptobrevin
OL: optic lobe
PDF: Pigment dispersing factor
PDFR: PDF receptor
PER: Period
PI: pars intercerebralis
PL: pars lateralis
PLP: posterior lateral protocerebrum
SAC: superior arch commissure
SCL: superior clamp
**s-DN**_**3**_: small dorsal neurons 3
SEZ: subesophageal zone
**s-LN**_**v**_: small ventrolateral neurons
SLP: superior lateral protocerebrum
SMP: superior medial protocerebrum
UAS: upstream activating sequence
ZT: Zeitgeber time
