# Supplementary material for: The Neuronal Circuit of the Dorsal Circadian Clock Neurons in Drosophila melanogaster
Source: Front Physiol. 2022 Apr 29;13:886432. doi: 10.3389/fphys.2022.886432 (PMC9100938; doi:10.3389/fphys.2022.886432)
Supplement: Supplementary file 2 [file Table1.DOCX]

**Table S1: Description of the used fly lines**

| **Fly line** | **Description** | **Reference** |
| --- | --- | --- |
| *w; Clk856-GAL4* | *GAL4* line driving in all clock neurons | Gummadova *et al.*, 2009 |
| *w; Clk4.1M-GAL4* | *GAL4* line driving in ~9 DN_1p_s | Zhang *et al.*, 2010a |
| *w; Clk9M-GAL4; Pdf-GAL80* | *Driving in 1-2 DN_2_* | Kaneko *et al.*, 2012 |
| *w; +; R16C05-GAL4* | *GAL4* line driving in 2 LN_d_s and 2 DN_1a_s | Pfeiffer *et al.*, 2008 |
| *R43D05-p65.AD; VT003234-DBD* | *Split-GAL4* line driving in one of the 2 DN_2_ | Sekiguchi *et al.*, 2020 |
| *R43D05-p65.AD; R93B11-DBD* | *Split-GAL4* line driving in the 2 DN_1a_ | Sekiguchi *et al.*, 2020 |
| *w; +; 10xUAS-myr::GFP* | Marking the addressed cells by expression of a membrane-bound myristoylated GFP (myrGFP) | Pfeiffer *et al.*, 2010 |
| y, w; PBac{20xUAS-6xGFP}VK00018/CyO, P{Wee-P.ph0}BaccWee-P20 | Marking the addressed cells by expression of a cytosolic myc-tagged GFP | Bloomington Cat# 52261 |
| UAS-nSyb::EGFP | Marking presynaptic sites (axonal terminals) | Zhang *et al.*, 2002 |
| w; UAS-DenMark::mCherry | Marking postsynaptic sites (dendrites) | Nicolaï *et al.*, 2010 |
| *y w;hs-mFlp5^MH12^/CyO;TM2/TM6B* |  | Shimosako *et al.*, 2014 |
| *y w;GlaBc/CyO;hs-mFlp^5MH3^/TM6B* |  | Shimosako *et al.*, 2014 |
| *hs-Flp^1^;+;FB2.0B^49b^* |  | Shimosako *et al.*, 2014 |
| *hs-Flp^1^;FB2.0B^260b^*;+ |  | Shimosako *et al.*, 2014 |
| UAS-*trans*-Tango  *UAS-myrGFP, QUAS-mtdTomato(3xHA); trans-Tango; +* | GFP expression in the *GAL4*-driver cells and expression of a second reporter (HA or mtdTomato) in putative postsynaptic neurons | Talay *et al.*, 2017 |

**References**

Gummadova, J. O., Coutts, G. A., and Glossop, N. R. J. (2009). Analysis of the Drosophila Clock Promoter Reveals Heterogeneity in Expression between Subgroups of Central Oscillator Cells and Identifies a Novel Enhancer Region. *J Biol Rhythms* 24, 353–367.
doi:10.1177/0748730409343890.

Kaneko, H., Head, L. M., Ling, J., Tang, X., Liu, Y., Hardin, P. E., *et al.* (2012). Circadian Rhythm of Temperature Preference and Its Neural Control in Drosophila. *Current Biology* 22, 1851–1857.
doi:10.1016/j.cub.2012.08.006.

Nicolaï, L. J. J., Ramaekers, A., Raemaekers, T., Drozdzecki, A., Mauss, A. S., Yan, J., *et al.* (2010). Genetically encoded dendritic marker sheds light on neuronal connectivity in Drosophila. *PNAS* 107, 20553–20558.
doi:10.1073/pnas.1010198107.

Pfeiffer, B. D., Jenett, A., Hammonds, A. S., Ngo, T.-T. B., Misra, S., Murphy, C., *et al.* (2008). Tools for neuroanatomy and neurogenetics in Drosophila. *PNAS* 105, 9715–9720.
doi:10.1073/pnas.0803697105.

Pfeiffer, B. D., Ngo, T.-T. B., Hibbard, K. L., Murphy, C., Jenett, A., Truman, J. W., *et al.* (2010). Refinement of Tools for Targeted Gene Expression in Drosophila. *Genetics* 186, 735–755.
doi:10.1534/genetics.110.119917.

Sekiguchi, M., Inoue, K., Yang, T., Luo, D.-G., and Yoshii, T. (2020). A Catalog of GAL4 Drivers for Labeling and Manipulating Circadian Clock Neurons in Drosophila melanogaster. *J Biol Rhythms*, 0748730419895154. doi:10.1177/0748730419895154.

Shimosako, N., Hadjieconomou, D., and Salecker, I. (2014). Flybow to dissect circuit assembly in the Drosophila brain. *Methods Mol Biol* 1082, 57–69.
doi:10.1007/978-1-62703-655-9_4.

Talay, M., Richman, E. B., Snell, N. J., Hartmann, G. G., Fisher, J. D., Sorkaç, A., *et al.* (2017). Transsynaptic Mapping of Second-Order Taste Neurons in Flies by trans-Tango. *Neuron* 96, 783-795.e4.
doi:10.1016/j.neuron.2017.10.011.

Zhang, L., Chung, B. Y., Lear, B. C., Kilman, V. L., Liu, Y., Mahesh, G., *et al.* (2010a). DN1p Circadian Neurons Coordinate Acute Light and PDF Inputs to Produce Robust Daily Behavior in Drosophila. *Current Biology* 20, 591–599.
doi:10.1016/j.cub.2010.02.056.

Zhang, Y. Q., Rodesch, C. K., and Broadie, K. (2002). Living synaptic vesicle marker: Synaptotagmin-GFP. *genesis* 34, 142–145.
doi:https://doi.org/10.1002/gene.10144.
