# Supplementary material for: The Neuronal Circuit of the Dorsal Circadian Clock Neurons in Drosophila melanogaster
Source: Front Physiol. 2022 Apr 29;13:886432. doi: 10.3389/fphys.2022.886432 (PMC9100938; doi:10.3389/fphys.2022.886432)
Supplement: Supplementary file 4 [file Table2.DOCX]

**Table S2: Primary and secondary antibodies used**

| **Antibody** | **Directed against** | **Source** | **Used final concentration** | **Host species** | **reference** |
| --- | --- | --- | --- | --- | --- |
| C7 anti-PDF monoclonal | NSELINSLLSLPKNMNDA-NH_2_ | DSHB | 1:2000 | Mouse | Deposited by J. Blau 2005 RRID: AB_760350 |
| anti-ITP | GGGDEEEKFNQ | H. Dircksen | 1:10000 | Rabbit | Dircksen et al., (2008)  RRID: AB_2315311 |
| anti-PER | full-length protein | R. Stanewsky | 1:2000 | Rabbit | Stanewsky et al., (1997) RRID: AB_ 2315105 |
| anti-CRY | His‐tagged form of full‐length dCRY | T. Todo | 1:1000  pre-absorbed on *cry^01^* embryos | Rabbit | Yoshii et al., (2008) RRID: AB_2314242 |
| anti-GFP | Recombinant full length protein corresponding to GFP | Abcam | 1:2000 | Chicken | RRID: AB_300798 |
| anti-mCherry | full-length protein mCherry | ThermoScientific | 1:2000 | Rat | RRID: AB_2536611 |
| anti-HA | YPYDVPDYA | Roche Diagnostics GmbH | 1:100 | Rat | Talay et al., (2017) RRID: AB_2687407 |
| anti-nc82 | Bruchpilot C-terminal aa 1227-1740 | Hofbauer | 1:50 | Mouse | Wagh et al., (2006) RRID: AB_2314866 |
| AlexaFluor488 (anti-chicken) | IgY (H+L) chicken | ThermoScientific | 1:200 | Goat | RRID: AB_2534096 |
| AlexaFluor555 (anti-mouse) | IgG (H+L) mouse | ThermoScientific | 1:200 | Goat | RRID: AB_141780 |
| AlexaFluor568 (anti-rat) | IgG (H+L) rat | ThermoScientific | 1:200 | Goat | RRID: AB_2534121 |
| AlexaFluor635 (anti-rabbit) | IgG (H+L) rabbit | ThermoScientific | 1:200 | Goat | RRID: AB_2536186 |
| AlexaFluor 635 (anti-rabbit) | IgG (H+L) rabbit | Thermo Scientific | 1:200 | Goat | RRID: AB_2536186 |
| AlexaFluor647 (anti-mouse) | IgG (H+L) mouse | ThermoScientific | 1:200 | Goat | RRID: AB_2535804 |

**References**

Dircksen, H., Tesfai, L. K., Albus, C., and Nässel, D. R. (2008). Ion transport peptide splice forms in central and peripheral neurons throughout postembryogenesis of Drosophila melanogaster. *J Comp Neurol.* 509, 23–41.
doi:10.1002/cne.21715.

Stanewsky, R., Frisch, B., Brandes, C., Hamblen-Coyle, M. J., Rosbash, M., and Hall, J. C. (1997). Temporal and Spatial Expression Patterns of Transgenes Containing Increasing Amounts of the Drosophila Clock Geneperiod and a lacZ Reporter: Mapping Elements of the PER Protein Involved in Circadian Cycling. *J. Neurosci.* 17, 676–696.
doi:10.1523/JNEUROSCI.17-02-00676.1997.

Talay, M., Richman, E. B., Snell, N. J., Hartmann, G. G., Fisher, J. D., Sorkaç, A., et al. (2017). Transsynaptic Mapping of Second-Order Taste Neurons in Flies by trans-Tango. *Neuron* 96, 783-795.e4.
doi:10.1016/j.neuron.2017.10.011.

Wagh, D. A., Rasse, T. M., Asan, E., Hofbauer, A., Schwenkert, I., Dürrbeck, H., et al. (2006). Bruchpilot, a Protein with Homology to ELKS/CAST, Is Required for Structural Integrity and Function of Synaptic Active Zones in Drosophila. *Neuron* 49, 833–844. doi:10.1016/j.neuron.2006.02.008.

Yoshii, T., Todo, T., Wülbeck, C., Stanewsky, R., and Helfrich-Förster, C. (2008). Cryptochrome is present in the compound eyes and a subset of Drosophila’s clock neurons. *J. Comp. Neurol.* 508, 952–966.
doi:10.1002/cne.21702.
